# Zinc-dependent turnover of ZIP3 transporter mRNA by trypanosome ZNK1

**DOI:** 10.1093/nar/gkag877

**Published:** 2026-09-12

**Authors:** Teresa Leão, Anna Trenaman, Michele Tinti, Gustavo Bravo Ruiz, Idálio J Viegas, Luisa M Figueiredo, Margarida Duarte, Ana Maria Tomás, David Horn

**Affiliations:** i3S—Instituto de Investigação e Inovação em Saúde, Universidade do Porto, Rua Alfredo Allen 208, 4200-135 Porto, Portugal; ICBAS—Instituto de Ciências Biomédicas Abel Salazar, Universidade do Porto, Rua de Jorge Viterbo Ferreira 228, 4050-313 Porto, Portugal; Faculty of Life Sciences, University of Dundee, Dow Street, Dundee DD1 5EH, United Kingdom; Faculty of Life Sciences, University of Dundee, Dow Street, Dundee DD1 5EH, United Kingdom; Faculty of Life Sciences, University of Dundee, Dow Street, Dundee DD1 5EH, United Kingdom; Faculty of Life Sciences, University of Dundee, Dow Street, Dundee DD1 5EH, United Kingdom; iMM – Instituto de Medicina Molecular, Universidade de Lisboa, Avenida Professor Egas Moniz, 1649-028, Lisboa, Portugal; iMM – Instituto de Medicina Molecular, Universidade de Lisboa, Avenida Professor Egas Moniz, 1649-028, Lisboa, Portugal; i3S—Instituto de Investigação e Inovação em Saúde, Universidade do Porto, Rua Alfredo Allen 208, 4200-135 Porto, Portugal; i3S—Instituto de Investigação e Inovação em Saúde, Universidade do Porto, Rua Alfredo Allen 208, 4200-135 Porto, Portugal; ICBAS—Instituto de Ciências Biomédicas Abel Salazar, Universidade do Porto, Rua de Jorge Viterbo Ferreira 228, 4050-313 Porto, Portugal; Faculty of Life Sciences, University of Dundee, Dow Street, Dundee DD1 5EH, United Kingdom

## Abstract

Like other cells, parasitic and other trypanosomatids sense Zn^2+^ and regulate Zn^2+^ transport, but the mechanisms involved remained unknown. Here, we identify a trypanosome RNA-binding protein that specifically eliminates *ZIP3* transporter mRNA in Zn^2+^-replete conditions. We first demonstrate that *Trypanosoma brucei ZIP3* mRNA abundance is subject to 3′-untranslated region (3′-UTR) and Zn^2+^-dependent negative control. A genome-wide RNA interference library screen, using a reporter associated with the *ZIP3* 3′-UTR, identifies Tb927.11.9510 as a candidate Zn^2+^-sensor, and we name this protein Zinc Nuclear Knuckles 1 (ZNK1) since it localizes to the nucleus and contains several Zn^2+^-knuckle motifs. ZNK1 is conserved among trypanosomatids, and a PIN domain suggests a ribonuclease-based mechanism. We use Cas9-editing to knockout ZNK1 and observe specific accumulation of *ZIP3* transcripts, and increased intracellular Zn^2+^, in *znk1* null cells. We validate ZNK1 as a *ZIP3* 3′-UTR-dependent negative regulator and identify a GU-repeat motif in the *ZIP3* 3′-UTR that is predictive of ZNK1-based negative control. In conclusion, ZNK1 eliminates *ZIP3* transporter mRNA in a Zn^2+^-dependent manner. We suggest that trypanosomatid ZNK1 is a highly selective zinc finger nuclease that binds GU-repeat motifs within *ZIP3* 3′-UTRs and degrades Zn^2+^ transporter mRNA only when the tandem sensor knuckle modules are coordinated with Zn^2+^.

## Graphical Abstract

**Graphical Abstract:**
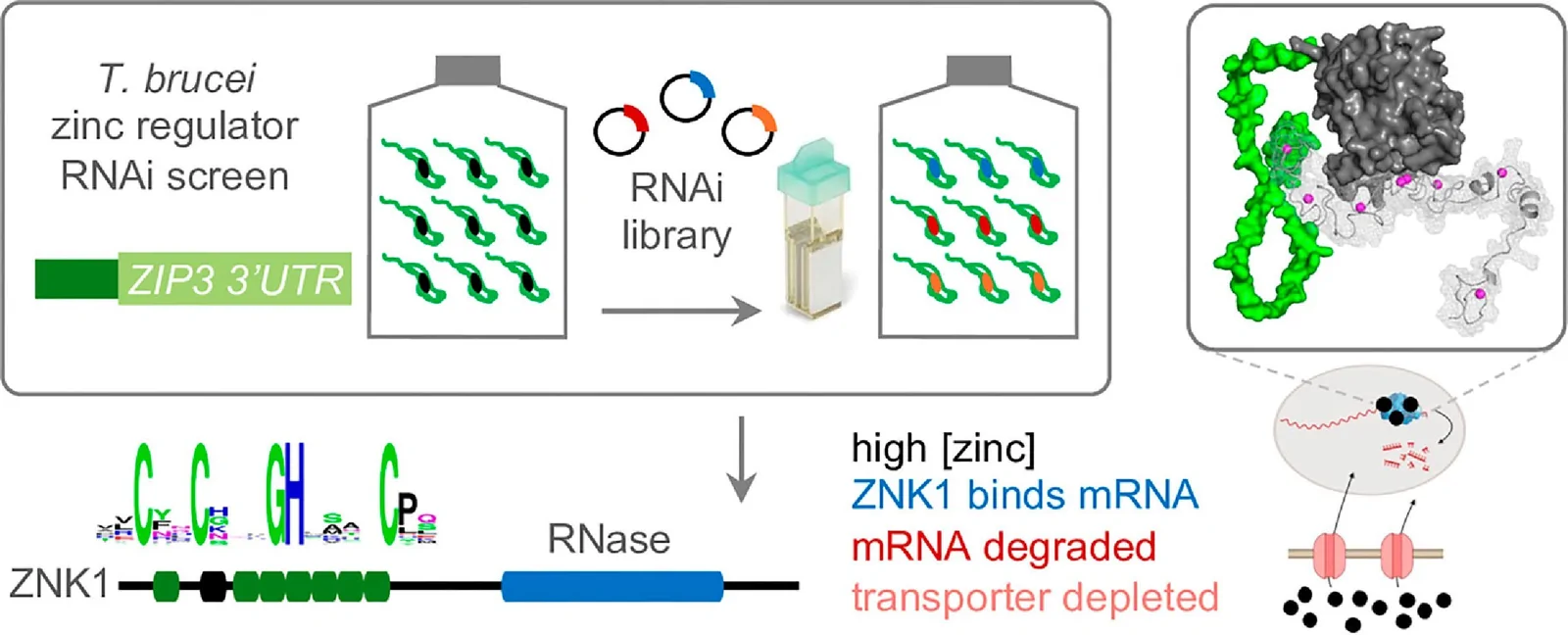

## Introduction

Parasitic protozoa such as trypanosomatids face constant challenges in acquiring essential metabolites from their environments, as they navigate distinct host niches with varying nutrient profiles. *Trypanosoma brucei*, for example, is transmitted by tsetse flies and causes African trypanosomiasis, a neglected tropical disease known as sleeping sickness in humans and a livestock disease known as nagana. These pathogens have evolved dedicated systems to sense fluctuations in nutrient and cofactor availability and to modulate acquisition and utilization. Because these parasites employ widespread polycistronic transcription, they depend almost exclusively on post-transcriptional control mechanisms, primarily mediated by RNA-binding proteins, to reprogram gene expression and for environmental adaptation [1]. Understanding how parasites detect and respond to these nutritional cues is critical not only for uncovering basic aspects of their biology, but also for identifying new targets for therapeutic intervention.

Zinc (Zn^2+^) is an essential micronutrient required for the structural and catalytic functions of numerous proteins involved in a wide range of cellular processes. Yet, because excess intracellular Zn^2+^ is cytotoxic, primarily due to protein mismetallation [2–4], cells must tightly regulate acquisition, distribution, and storage [5]. This is typically achieved through the coordinated action of membrane transporters, including cellular and organellar importers and exporters, metal-binding proteins, and regulatory factors [6].

Despite the importance of zinc homeostasis for cell viability, the molecular mechanisms by which trypanosomatids sense and regulate zinc availability remain largely unexplored. In many systems, zinc is carried into cells by Zrt/Irt-like (ZIP) proteins, zinc-regulated and iron-regulated transporters. These proteins translocate zinc and other divalent metals from the extracellular environment or between intracellular compartments and the cytosol [7, 8]. To date, a single ZIP has been characterized as a zinc-regulated zinc transporter in trypanosomatids, *Li*ZIP3 in *Leishmania infantum*, another parasite, and the causative agent of visceral leishmaniasis [9]. Genome annotation predicts the presence of ZIP family members in other trypanosomatids [10], including orthologues of *Li*ZIP3, but little is known about their regulation. Indeed, factors that mediate zinc-responsive gene expression have not been identified in any trypanosomatid.

Here, we dissect how *T. brucei* coordinates the expression of ZIP3 orthologs in response to changes in zinc availability. We first characterize the regulation of *ZIP3* and find that its mRNA is subject to post-transcriptional repression under zinc-replete conditions, by a mechanism involving the *ZIP3* 3′-UTR. We then devise a genome-wide RNA interference library screen [11] using a zinc-responsive reporter under the control of the *ZIP3* 3′-UTR. We identify ZIP-family members, including ZIP3, as likely effectors of zinc accumulation and a putative regulatory RNA-binding protein, which we name Zinc Nuclear Knuckles 1 (ZNK1); the protein is nuclear-localized and contains several Zn^2+^-knuckle motifs as well as a putative ribonuclease domain. We find a GU-repeat motif in the *ZIP3* 3′-UTR that is predictive of negative control by ZNK1 and show that ZNK1 is a negative regulator of *ZIP3* expression. Indeed, CRISPR-Cas9-mediated ZNK1 knockout led to specific accumulation of *ZIP3* transcripts in zinc-replete conditions, suggesting that ZNK1 mediates zinc-dependent mRNA degradation. Together, our results reveal a novel post-transcriptional regulatory mechanism by which trypanosomes maintain zinc homeostasis. Thus, ZNK1 is zinc-dependent and displays highly selective control of *ZIP3* mRNA turnover.

## Materials and methods

### *T. brucei* growth and manipulation

Bloodstream form Lister 427 *T. brucei* and derivatives, including 2T1 [12], were cultured in HMI-11 (Gibco) supplemented with 10% foetal bovine serum (Sigma) in a humidified incubator at 37°C with 5% CO_2_. Genetic manipulations were carried out by electroporation using a Nucleofector (Lonza), with cytomix for routine transfections and a human T-cell kit (Lonza) for high-efficiency library transfections [11]. Selection of recombinant bloodstream form clones was carried out by the addition of phleomycin, G418, puromycin, hygromycin and/or blasticidin at 2, 2, 2, 2.5, and 10 μg/ml, respectively; clones were subsequently maintained in 1, 1, 1, 1, and 2 μg/ml of each antibiotic, respectively. Tetracycline, N,N,N',N'-tetrakis(2-pyridinylmethyl)-1,2-ethanediamine (TPEN), deferoxamine (DFO), and actinomycin D were obtained from Sigma. Tetracycline was used at 1 μg/ml to induce RNAi knockdown or inducible expression of Cas9. TPEN [13] and DFO [14] were applied at 10 µM, equivalent to ~2 × EC_50_. Transcription was inhibited by treating cells with 5 µg/ml of actinomycin D. Cell density was determined using a haemocytometer.

### Phylogenetic analysis

Alignment and phylogenetic reconstructions were performed using the function ‘build’ of ETE3 3.1.3 [15].

### Plasmid construction

To generate the Neo^R^  *ZIP3* 3′-UTR reporter construct, a PCR fragment corresponding to the full 1082 bp inter-CDS region of *ZIP3* was amplified from wild-type (WT) *T. brucei* genomic DNA using primers P9, carrying a BamHI site, and P10, carrying a BglII site. The Tub-Neo-BSD-TK plasmid [16] was digested with BamHI and BglII to remove a BSD-TK_Ald 3′-UTR fragment, and the PCR product was inserted into those sites. The 1082 bp region was verified by Sanger sequencing and was found to be identical to the Tb427_110096600-700 (*ZIP3*) intergenic sequence. To generate the mutant *ZIP3* 3′-UTR reporter plasmids MUT1-3, which were designed prior to UTR re-annotation [16], the Neo_3′-UTR plasmid was amplified by overlap-extension PCR using Phusion high-fidelity polymerase, using the following primer pairs: MUT1:Fw and MUT1:Rv; MUT2:Fw and MUT2:Rv; MUT3:Fw and MUT3:Rv. The modified UTR-reporter cassettes were verified by Sanger sequencing. *T. brucei* were transfected with the recombinant plasmids following digestion with PmeI. To generate the *ZNK1* RNAi construct (pRPa^iSL^_11.9510), a 517 bp Tb927.11.9510 CDS fragment was selected using the RNAit tool [17]. This fragment was amplified by PCR from *T. brucei* genomic DNA and cloned in the tetracycline-inducible RNAi construct, pRPa^iSL^ [18]. The forward primer (P5) carried ApaI and KpnI sites, and the reverse primer (P6) carried XbaI and BamHI sites for cloning in either orientation. The resulting pRPa^iSL^_11.9510 plasmid was verified by diagnostic restriction digestion and Sanger sequencing, then linearized with AscI prior to transfection into *T. brucei*. To generate the N-terminal 6 × myc ZNK1 tagging construct, a 1134 bp fragment corresponding to the 5′ region of the Tb927.11.9510 gene was amplified by PCR from *T. brucei* genomic DNA using primers that introduced an AvrI restriction site at the 5′ end (P7) and a BamHI site at the 3′ end (P8). The PCR product was digested with AvrI and BamHI and cloned into the corresponding sites in pNAT_BSD^6xmyc^ [18]. The resulting plasmid was verified by restriction digestion and Sanger sequencing. *T. brucei* were transfected with this plasmid following digestion with SalI.

### RT-qPCR

Total RNA was extracted from bloodstream-form *T. brucei* cells using the RNeasy kit (Qiagen), including on-column DNase digestion, according to the manufacturer’s instructions. First-strand cDNA was synthesized from 1–2 µg of DNase-treated RNA using M-MLV™ Reverse Transcriptase (Sigma) and oligo-dT primers. Quantitative real-time PCR was performed on a QuantStudio 3 (Thermo Fisher Scientific) using a Luna^®^ Universal qPCR Master Mix (New England Biolabs) and gene-specific primers for Tb927.11.8990/*ZIP3a* (P15 + P16, 86 bp amplicon), Tb92711.9000–9030/*ZIP3* (P17 + P18, 94 bp amplicon), Tb927.11.9510/*ZNK1* (P19 + P20, 137 bp amplicon), Tb927.5.4140 (P21 + P22, 176 bp amplicon), Neo^R^/*NPTII* (P23 + P24, 114 bp amplicon), and the tubulin reference gene (P25 + P26, 209 bp amplicon). Reactions were run in technical triplicate, and a no-RT control was included to confirm the absence of genomic DNA contamination. Melt-curve analysis was used to verify amplification specificity and yielded a single peak for all six targets. Relative mRNA abundance was calculated using the 2^−ΔΔCt^ method, normalizing Ct values to the reference gene and expressing data relative to the t = 0 control.

### Dose-response assay

*T. brucei* were seeded at 2 × 10^3^ cells/ml in 96-well plates containing increasing concentrations of G418 in quadruplicate. Sixty-six hours later, 20 µl of 0.125 mg/ml resazurin was added. Plates were incubated for a further 6 h prior to measuring cell viability using a plate reader with the following settings: λexc 530 nm and λem 585 nm. Data were converted to ‘% Survival’ by subtracting experimental values from the average of HMI-11 (media)-only control, followed by calculation of proportion of *T. brucei*-only control. The half-maximal effective concentration (EC_50_) was derived from nonlinear regression analysis, using the ‘Sigmoidal dose-response (variable slope)’ function in GraphPad Prism 8 (GraphPad Software, Inc., La Jolla, CA, USA).

### RNAi target sequencing (RIT-seq)

The RIT-seq screen was carried out essentially as described [11, 19], except that cells were selected in 8 μg/ml G418 for seven or ten days prior to extraction of genomic DNA. High-throughput sequencing was on a DNBseq platform (Illumina) at the Beijing Genomics Institute (BGI). The sequencing data analysis pipeline was adapted from [11]. The FASTQ files with forward and reverse paired-end reads (four technical replicates for each sample) were concatenated and aligned to the reference genome v46 of *T. brucei* clone TREU927 downloaded from TriTrypDB [10] using Bowtie2 (2.3.5) [20] with the ‘very-sensitive-local’ preset alignment option. The alignments were converted to BAM format, reference sorted, and indexed with SAMtools (1.9) [21]. The alignments were deduplicated with the Picard tools package using the MarkDuplicates function (http://broadinstitute.github.io/picard/); to minimize the potential for over-representation of the shortest RIT-seq fragments. Alignments with properly paired reads were extracted with SAMtool view using the -f 2 option and parsed with a custom Python script to extract the paired reads containing the index sequence (GTGAGGCCTCGCGA) in forward or reverse complement orientation. The genome coverage of the aligned reads was extracted from the bam files using deeptools (3.5) in bedGraph format [22]. The bedGraph read-mapping files were visualized with the svist4get Python package [23].

### Immunofluorescence microscopy

Immunofluorescence microscopy was conducted on *T. brucei* cells fixed in 1% formaldehyde, dried onto slides in 1% (w/v) BSA, and permeabilized with 0.5% (v/v) Triton X-100. Primary mouse α-cmyc 9B11 (Cell Signalling Technology) antibody was used at 1:5000 dilution, and secondary α-mouse Alexa 488 (Invitrogen) antibody was used at 1:2000. Cells were mounted in Vectashield (Vector Laboratories) containing DAPI (4′,6-diamidino-2-phenylindole). Images were acquired using a AxioImager Z1 microscope (Carl Zeiss) and analysed with ImageJ. Excitation and emission wavelengths were 365 – 445–450 nm, respectively, for DAPI and 440–70 – 525/50 nm, respectively, for Alexa 488.

### Protein blotting

Cells for western blotting analysis were lysed in 4% (w/v) SDS, 50 mM Tris–HCl pH 7.4 without heating, and extracts were run in 12% SDS–PAGE gels. Western blotting was performed according to standard protocols, with primary rabbit α-NPTII (Fitzgerald Industries International) and mouse α-EF1α CBP-KK1 (Merck Millipore) used at 1:5000 dilution. Detection was performed with the Bio-Rad Clarity™ chemiluminescence kit (Bio-Rad) using a Chemidoc (Bio-Rad). Images were analysed using ImageLab (Bio-Rad).

### CRISPR–Cas9-mediated gene knockout

CRISPR–Cas9-mediated disruption of *ZNK1* was performed essentially as described [24]. Briefly, we designed a 20-nt single-guide RNA (sgRNA) targeting the *ZNK1* coding sequence. Complementary oligonucleotides (P11/P12) were annealed and ligated to the pT7sgRNA vector. The resulting plasmid (pT7sgRNA_11.9510) was verified by Sanger sequencing, linearized with NotI, and transfected into 2T1^T7-Cas9^ cells that express tetracycline-inducible Cas9. Cas9 expression was induced in the resulting clones, which were then transfected with an NPTII gene cassette flanked by sequences homologous to the *ZNK1* locus, which was amplified using primers P13 + P14. Edited clones were recovered using G418 selection. Precise *ZNK1* knockout was confirmed by diagnostic PCR and whole genome sequencing.

### Whole genome sequencing and analysis

*T. brucei* genomic DNA was extracted using DNazol (ThermoFisher), following the manufacturer’s instructions. Genome sequencing for *ZNK1* null and control strains was performed on a DNBseq platform (BGI); 3.5 Gbp/sample, 150 bp read length. Initial quality control of FASTQ files was performed using FastQC (https://www.bioinformatics.babraham.ac.uk/projects/fastqc/), followed by adapter trimming and quality filtering with Fastp (0.20.0) [25]. Processed reads were aligned to the TREU927 genome assembly (TriTrypDB v68) [10] using Bowtie2 (2.3.5) [20] with ‘–very-sensitive-local’ parameters. The resulting alignments were processed with SAMtools (1.9) [21] for sorting and indexing. Bam files were transformed to bigWig track files using bamCoverage from DeepTools (3.5) [22] with –binSize 5 and –normalizeUsing RPKM. The linear coverage visualization was performed with the pyGenomeTracks [26] Python package. Bam files from parental and derived clones were analysed using bamcompare from DeepTools (3.5) [22] with –bin 200, smooth 500, –operation log2, –normalizeUsing RPKM –extendReads, –scaleFactorsMethod None, and –outFileFormat bedgraph. The circular fold-change visualization of the bedgraph files was performed with the pyCirclize (1.6) Python package (https://github.com/moshi4/pyCirclize).

### Transcriptomics

RNA-seq analysis was conducted on *znk1* null and control strains. Total RNA was extracted using a Qiagen RNeasy kit in triplicate according to the manufacturer’s instructions. Sequencing was performed on a HiSeq platform (Ilumina) at the Beijing Genomics Institute. Initial quality control of FASTQ files was performed using FastQC, followed by adapter trimming and quality filtering with Fastp (0.20.0) [25]. Processed reads were aligned to the reference TREU927 genome or Lister 427_2018 genome (TriTrypDB v68) [10] using Bowtie2 (2.3.5) [20] with ‘–very-sensitive-local’ parameters. The resulting alignments were processed with SAMtools (1.9) [21] for sorting and indexing, and PCR duplicates were marked using Picard MarkDuplicates (2.22.3) [27]. Read counts per coding sequence were quantified using featureCounts (1.6.4) [28] with parameters accounting for multi-mapping reads (-M) and overlapping features (-O) and configured to count only reads where both ends were mapped (-B) and to exclude chimeric reads that mapped to different chromosomes (-C). The datasets were then used as input for differential expression analysis using the edgeR package (3.28) [29] in R (3.6.1). We retained only genes that had a minimum count of 10 reads in at least one sample and a minimum total count of 30 reads across all samples. The cqn package (1.32) [30] was used to compute gene length and GC content bias. The resulting offsets were incorporated into the DGEList object before computing dispersions. We fitted our data to a generalized linear model (GLM) using the quasi-likelihood (QL) method via glmQLFit and performed differential expression testing using the quasi-likelihood F-test through the glmQLFTest function. The output from this statistical test was then processed using the topTags function, which extracted the complete set of test results. We configured topTags to return all tested genes (n = Inf) without applying any sorting (sort.by = ‘none’), ensuring that the original gene order was preserved in the output. For multiple testing correction, we employed the Benjamini–Hochberg (BH) procedure (adjust.method = ‘BH’) to control the false discovery rate.

### Metal accumulation assays

Approximately 5 × 10^7^ WT and *znk1* null *T. brucei* cells, including samples supplemented with 100 µM ZnCl_2_ for 2 h, were harvested by centrifugation at 1000 × *g* for 10 min at 4°C. Cells were washed once in ice-cold PBS with 0.1 mM EDTA and once with PBS, then dried at 67°C and stored at −80°C. Prior to analysis, samples were digested in 100 µl HNO_3_ plus 900 µl ultra-pure water. Zinc and iron were then quantified by inductively coupled plasma-mass spectrometry using an iCAP^TM^ Q ICP-MS (Thermo Fisher Scientific).

### *In silico* analysis of motif enrichment in the *ZIP3* 3′-UTR

Motif enrichment analysis was performed by Multiple Em for Motif Elicitation (MEME) [31] using sequences 1 kb downstream from the stop codons of *ZIP3* (Tb927.11.9000) orthologous genes from multiple trypanosomatids (Supplementary Data 1, sheet 6), one from each species (data from tritrypdb.org) [10]. MEME analyses were run to identify any number of 8-b motif repetitions in the given strand only, with otherwise default conditions in classic mode. The most enriched GUGUGUG(U/C) motif was then used to determine motif frequency across the ‘427’ genome (*T. brucei* Lister 427_2018 v68, from TryTrypDB) [32], using data from the sense strand only, and considering a *P*-value < 3e^−5^ (score > 14). The motifs were then clustered into blocks at least 40 bp apart. The results were visualized using ggplot2 (https://ggplot2.tidyverse.org) and the circlize package (version 0.4.16).

### Oligonucleotides

Details of oligonucleotides used for plasmid construction, RT-qPCR, *ZNK1* knockout, and *ZIP3* 3′-UTR mutagenesis can be found in Supplementary Data 1, sheet 7.

## Results

### *T. brucei ZIP3* mRNA turnover is zinc- and 3′-UTR-dependent

The *Leishmania infantum* zinc importer *Li*ZIP3 (LINF_280025700–800) is negatively regulated at the mRNA level in response to zinc [9] and we reasoned that the *T. brucei* ortholog would be similarly regulated. The most closely related ortholog of *Li*ZIP3 in *T. brucei* is encoded by a syntenic tandem cluster on chromosome 11 (Tb927.11.9000–Tb927.11.9030 in the reference strain, Tb427_110096600–110097800 in our experimental strain, https://tritrypdb.org). We refer to these genes as *Tb*ZIP3. Both *ZIP3* tandems are preceded by a gene encoding a diverged paralog (Tb927.11.8990, Tb427_110096500; *Tb*ZIP3a), while the *ZIP3* tandem in our experimental strain is followed by two genes encoding diverged paralogs (Tb427_110097900–8000; *Tb*ZIP3b-c). A phylogenetic analysis (Supplementary Fig. S1) shows the relationship among these ZIP3 paralogs and orthologs and also includes reported iron transporters, LIT1 [33] and *Tc*IT [34].

**Figure 1.**
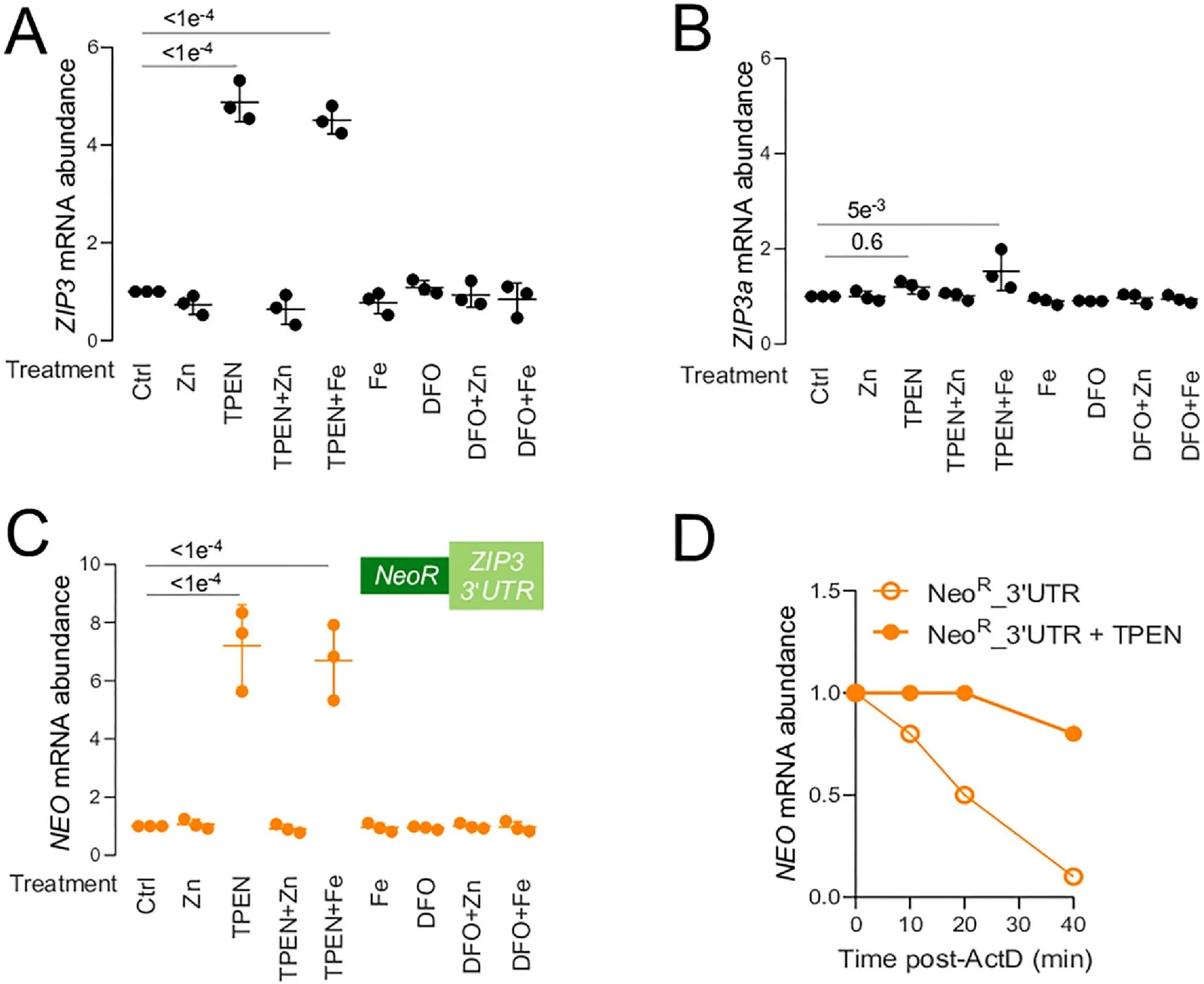
*Trypanosoma brucei ZIP3* mRNA is subject to zinc- and 3′-UTR-dependent turnover. The plots show RT-qPCR analysis to assess the abundance of *ZIP3* (**A**) or *ZIP3a* (**B**) transcripts in *T. brucei* grown in zinc-sufficient (Ctrl), zinc/iron-surplus (Zn/Fe, 100 µM, 1 h), or zinc/iron-limited medium; TPEN (for zinc, 10 µM, 2 h) and DFO (for iron, 10 µM, 2 h). Data represents fold change in expression relative to the control (Ctrl), normalized to β-tubulin transcript abundance. *P*-values were derived using one-way ANOVA tests with Sidak multiple comparisons. Mean ± SD from three independent experiments are shown. (**C**) The plot shows RT-qPCR analysis to assess the abundance of the Neo^R^ reporter transcript under the control of the *ZIP3* 3′-UTR. Other details as in panels (A, B). (**D**) The plot shows RT-qPCR analysis to assess the Neo^R^  *ZIP3* 3′-UTR reporter transcript after inhibition of transcription with actinomycin D (ActD, 5 µg/ml) in control or TPEN-treated cells. *n* = 1. Other details as in panels (A, B).

Our first step was to determine whether *ZIP3* or *ZIP3a* expression responded to zinc or iron, two of the most common metals transported by ZIP family transporters [35–37]. Bloodstream form (BSF) *T. brucei* were grown for 1–3 h in zinc-sufficient (Ctrl), zinc/iron-surplus (Zn/Fe), or zinc/iron-limited medium generated by treatment with the chelators TPEN (for zinc) or DFO (for iron), and expression of *ZIP3* and *ZIP3a* mRNA was then assessed using RT-qPCR. *ZIP3* displayed robust and significantly increased expression in zinc-limited medium, with transcript levels increased >4-fold relative to expression in control or zinc-surplus medium (Fig. 1A). This response was zinc-specific, since only addition of zinc, but not iron, abrogated TPEN-dependent upregulation (TPEN + Zn), while supplementation with iron had no significant effect (Fig. 1A). In similar assays, *ZIP3a* transcripts showed a significant, but relatively moderate 50% increased abundance in TPEN + Fe (Fig. 1B), perhaps indicating that *ZIP3a* is moderately responsive to metal ion availability.

In trypanosomatids, elements regulating gene expression are often embedded within the 3′-UTR, and the *ZIP3* 3′-UTR (Tb927.11.9000–9020) was notably linked to reduced expression in a recent massive parallel reporter assay [16]. To assess the *ZIP3* 3′-UTR, we generated a reporter strain expressing a G418 resistance gene (Neo^R^) under the control of this UTR, integrating the reporter cassette at the *tubulin* locus. To determine whether the 3′-UTR impacted Neo^R^ expression, we subjected the reporter strain to the same metal supplementation/limitation conditions detailed above. RT-qPCR analysis to assess Neo^R^ expression revealed a response to zinc that mirrored the *ZIP3* response, whereby Neo^R^ mRNA expression was significantly increased in the presence of TPEN, with this effect reversed by the addition of zinc but not by the addition of iron (Fig. 1C). Sensing through the *ZIP3* 3′-UTR thus likely enables *T. brucei* parasites to adapt to zinc availability.

To determine whether the *ZIP3* 3′-UTR drives mRNA turnover in the presence of zinc, we used actinomycin D (ActD) to inactivate transcription and again assessed reporter mRNA abundance in zinc-sufficient or limiting medium. We observed that in zinc-sufficient conditions the transcript decayed with a half-life of ~20 min, whereas under zinc-limiting conditions (TPEN), the transcript was relatively stable (Fig. 1D and Supplementary Fig. S2). These results indicated that the *ZIP3* transcript was indeed more rapidly turned over in the presence of zinc.

### A genome-wide screen for loss of *ZIP3* 3′-UTR-based negative control

We next sought to identify the regulatory factor or factors responsible for *ZIP3* mRNA turnover. We demonstrated above that a *Neo^R^* transcript under the control of the *ZIP3* 3′-UTR is unstable in zinc-sufficient medium and is stabilized when zinc is limiting. We, therefore, designed a genome-wide RNA interference (RNAi) target sequencing (RIT-seq) screen for knockdowns that increase Neo^R^  *ZIP3* 3′-UTR reporter expression in zinc-sufficient medium. Although genome-wide RIT-seq screens have been used widely for unbiased forward genetics and gene discovery, few screens have been used to identify factors operating via 3′-UTRs [38, 39].

**Figure 2.**
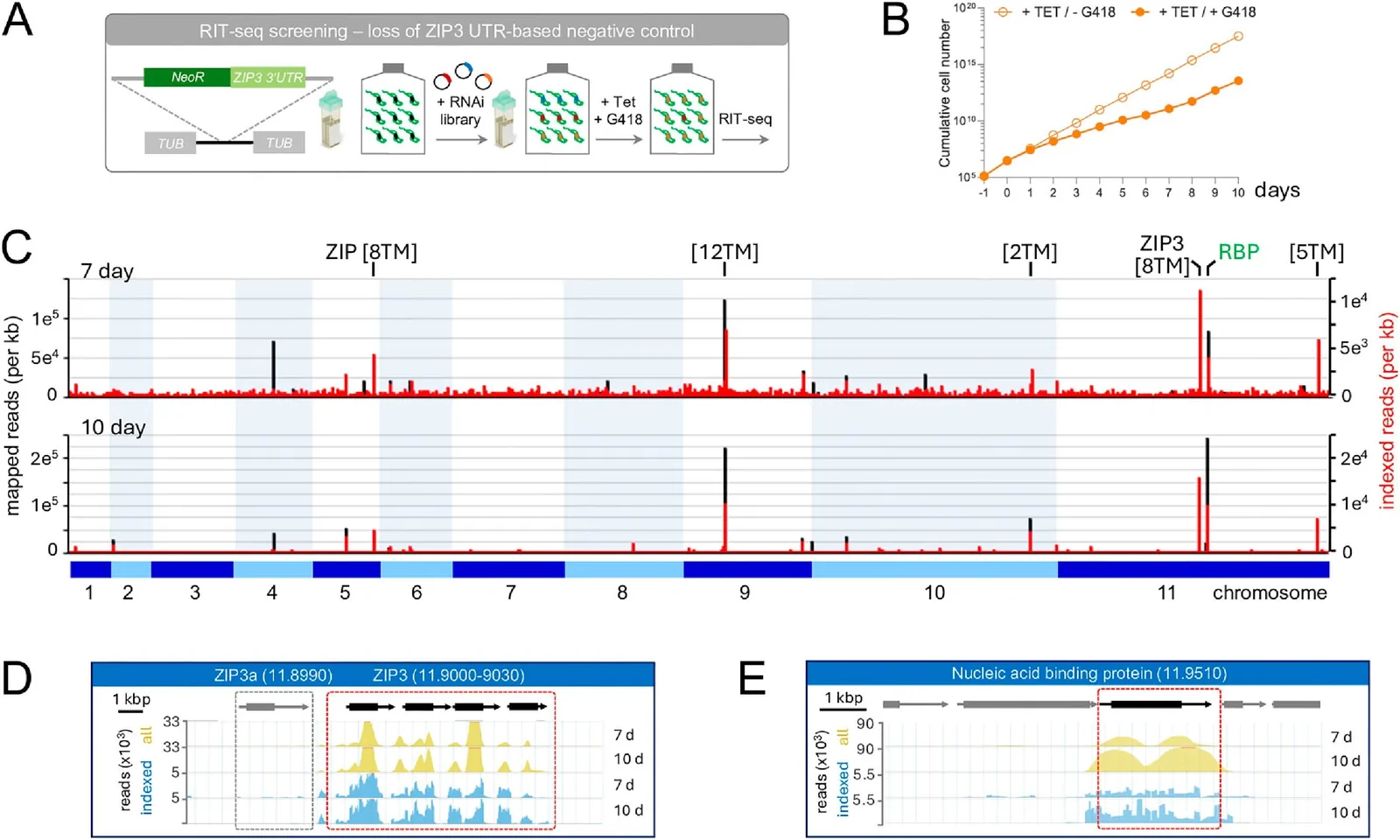
A genome-wide screen for loss of *ZIP3* 3′-UTR-based negative control. (**A**) Schematic representation of the genome-scale RNAi (RIT-seq) screen. *T. brucei* expressing the zinc-responsive Neo^R^  *ZIP3* 3′-UTR reporter were used to assemble an RNAi library. RNAi knockdown was induced with tetracycline (Tet, 1 µg/ml), G418 selection for loss of *ZIP3* 3′-UTR-based negative control was applied 24 h later, and genomic DNA was extracted for RIT-seq profiling. (**B**) The plot shows cell density monitored during RNAi library selection with G418. Two parallel libraries were induced with tetracycline, one was selected with G418, and the other served as the control. DNA was collected from the G418-selected library after 7 and 10 days. (**C**) Genome-scale RIT-seq profiles show enriched RIT-seq fragments. Data for both 7 and 10 days are shown. Black peaks represent total mapped reads per kilobase, whereas red peaks represent reads that incorporate an index sequence from the RNAi plasmid library. The putative RNA-binding protein (RBP), Tb927.11.9510, is indicated. Number of transmembrane (TM) domains predicted using TMHMM are also indicated for further hits (see Supplementary Data 1, sheet 1). (**D**) RIT-seq profiles show enriched RIT-seq fragments at the *ZIP3/3a* locus (*ZIP3*, red dashed box; *ZIP3a*, grey dashed box). Total reads (yellow) and indexed reads (blue) are shown for both 7 day (7 d) and 10 day (10 d) samples. (**E**) RIT-seq profiles as in panel (D) but for the Tb927.11.9510 locus.

To establish the RNAi library, we first transformed 2T1-T7 cells [12] with the zinc-sensitive Neo^R^  *ZIP3* 3′-UTR reporter construct; these cells also express the tetracycline repressor and T7 RNA polymerase, which allow for inducible expression of the RNAi library [11] (Fig. 2A). We then determined the effective concentration of G418 to inhibit the growth of these cells by 50% (EC_50_) to be 4 µM (Supplementary Fig. S3), which reflects Neo^R^ expression in these cells. The Neo^R^  *ZIP3* 3′-UTR reporter strain was then used to generate the RNAi library [11].

To run the screen, the RNAi library was induced with tetracycline and grown in the presence of G418 at 8 µM (2 × EC_50_). Cell density was monitored for 10 days under these conditions and compared to a parallel control culture grown without G418 selection (Fig. 2B). G418 selection impaired library growth as expected, and we harvested cells after 7 and 10 days when G418 had reduced cell number >1300-fold and >8800-fold relative to the control culture, respectively (Fig. 2B). Genomic DNA was extracted from the harvested cells, and DNA libraries were generated by PCR amplification of the RNAi target fragments. The amplicons were deep sequenced, and reads were mapped to the *T. brucei* reference genome. The genome-scale maps reveal substantial enrichment of several RIT-seq fragments, validated by the presence of flanking index sequences present in the plasmid library (Fig. 2C, Supplementary Data 1, sheet 1). Notably, those enriched RIT-seq fragments were very similar after either 7 or 10 days of library selection, but with fewer background reads after 10 days; we considered those top six hits, on chromosomes 5 and 9–11 that registered >3000 reads incorporating the index sequence from the RNAi plasmid library in both datasets (labelled red bars in Fig. 2C).

Five of these hits identified genes with between two and twelve predicted transmembrane domains, including *ZIP3* (Fig. 2D, red dashed box). ZIP3 knockdown likely depletes intracellular pools of zinc, essentially mimicking TPEN treatment (see Fig. 1C), and this probable indirect activation of the *ZIP3* 3′-UTR reporter by ZIP3 knockdown provided excellent validation for our screen. A similar rationale applies to Tb927.5.4140, which is also annotated as a ZIP-family member (Supplementary Fig. S4A). Unlike *ZIP3*,however, Tb927.5.4140 expression was not sensitive to zinc availability in the growth medium (Supplementary Fig. S4B); this ZIP may provide a non-redundant function, by mediating zinc transport from intracellular organelles to the cytosol, for example. Notably, *ZIP3a* did not register as a hit in the screen (Fig. 2D, grey box), suggesting a distinct role, and/or regulation, for this divergent paralog. The remaining three putative transmembrane proteins included a Major Facilitator Superfamily (MFS) transporter (Tb927.9.6360–80), previously shown to sensitize *T. brucei* to the aminoglycosides G418 and paromomycin [40], suggesting a role in G418 transport rather than zinc transport in this case. We suspect that the remaining two predicted transmembrane proteins also play roles in either G418 or zinc transport.

We were particularly interested in identifying the regulatory factor or factors responsible for *ZIP3* mRNA turnover, and the third top hit in the RIT-seq screen (Tb927.11.9510/Tb427_110103100) was annotated as a putative nucleic acid binding protein (Fig. 2E). This gene is on the same chromosome as the *ZIP3* array, but these loci are in distinct polycistrons and are separated by 116 kbp. Tb927.11.9510 presented an excellent candidate *ZIP3* regulator.

### Tb927.11.9510/ZNK1 is a conserved probable zinc sensor

**Figure 3.**
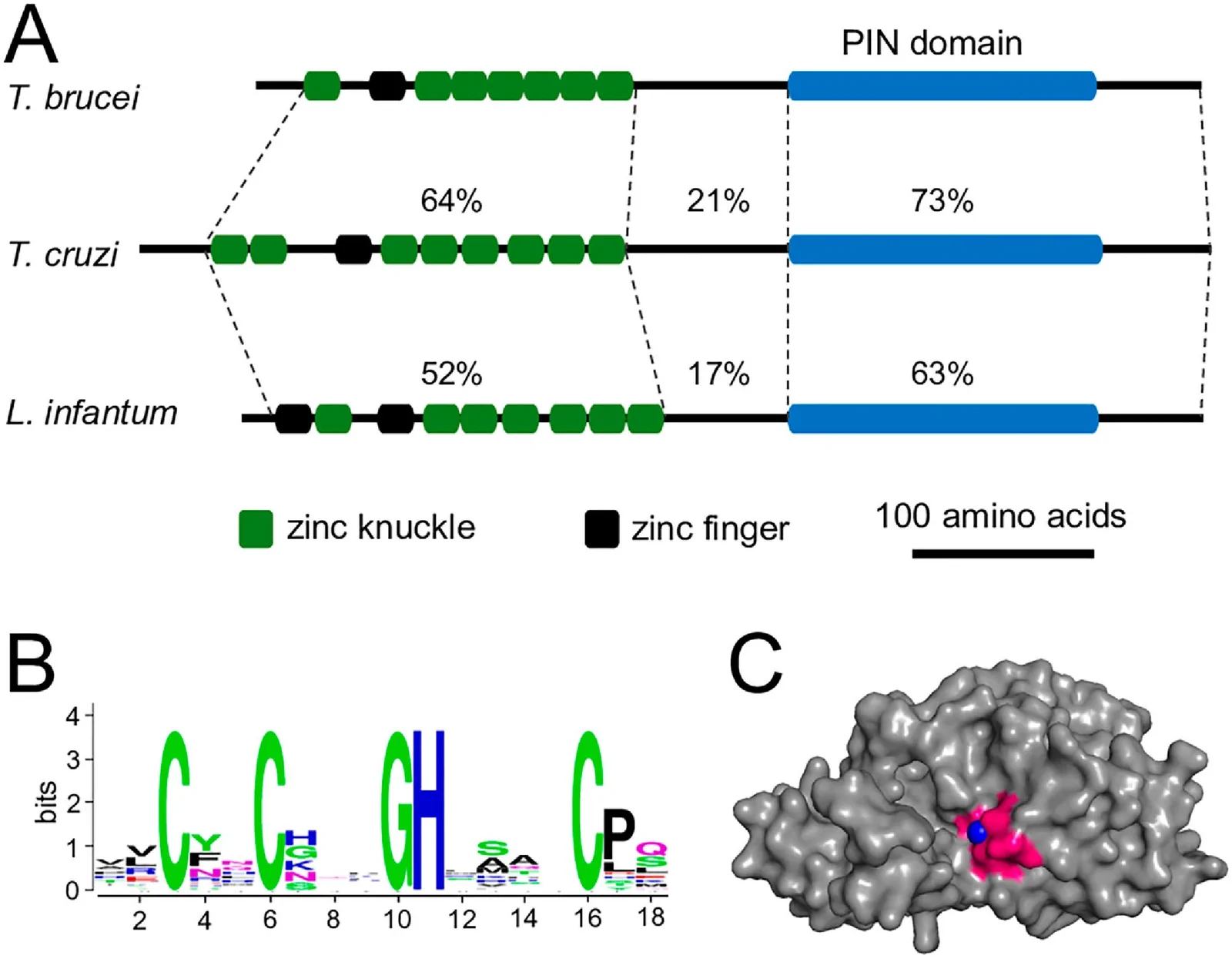
Tb927.11.9510/ZNK1 is a conserved probable zinc sensor. (**A**) Schematic representation of the protein encoded by Tb927.11.9510, and the orthologs in *T. cruzi* (TcCLB.511589.110) and *Leishmania infantum* (LINF_360040200). Zinc knuckle motifs (CxxCxxxGHxxxxC, green), zinc finger motifs (CCHC, black), and the putative RNase PIN domain (blue) are shown in each protein. Percentage values indicate identity with *T. brucei* ZNK1. (**B**) The sequence logo shows conserved residues in zinc knuckle motifs (*n* = 22) from the three proteins shown in panel (A). (**C**) The AlphaFold model (https://alphafoldserver.com/) of the *T. brucei* PIN domain (residues 288–516) was visualized using PyMOL and shows four Asp residues (D^294^, D^416^, D^441^, and D^443^; pink) coordinating Mg^2+^ (blue) at the putative RNase active site; interface predicted template modelling (ipTM) score = 0.87, predicted template modelling (pTM) score = 0.88.

*In silico* analysis of the protein encoded by the *Tb927.11.9510* gene revealed seven zinc knuckle motifs (CxxCxxxGHxxxxC) and one canonical ‘CCHC’ zinc finger motif, followed by a diverged ‘linker’ region and a PIN (PilT N-terminus) potential ribonuclease (RNase) domain (Fig. 3A), features entirely consistent with a zinc-sensing RNA-binding protein that can degrade *ZIP3* mRNA. Indeed, we find ZNK1 to be highly conserved among the parasitic trypanosomatids, *T. brucei, T. cruzi*, and *L. infantum* (Fig. 3A), consistent with a conserved role in zinc-sensing and *ZIP3* mRNA turnover. Zinc fingers and zinc knuckles are small cysteine/histidine-rich modules that coordinate Zn^2+^ ions and are typical of metal-dependent RNA-binding proteins, linking metal binding to conformational change that stabilizes the protein and/or facilitates RNA interaction [41, 42]. These motifs are also broadly conserved in number and spacing (Fig. 3A) and consensus sequence (Fig. 3B) in the ZNK1 orthologs; *T. cruzi* ZNK1 incorporates one additional zinc knuckle motif while *L. infantum* ZNK1 incorporates one additional CCHC-type zinc finger motif.

The PIN domain likely adopts an RNase-like fold, with conserved acidic residues coordinating a divalent metal ion essential for nucleic acid cleavage [43]. Indeed, an AlphaFold model of the *T. brucei* PIN domain supports this view (Fig. 3C). Since further evidence indicated a nuclear localization for the protein encoded by Tb927.11.9510 [44, 45] (also see below), we name this protein Zinc Nuclear Knuckles 1 (ZNK1).

### Specific accumulation of *ZIP3* transcripts in *znk1* null cells

**Figure 4.**
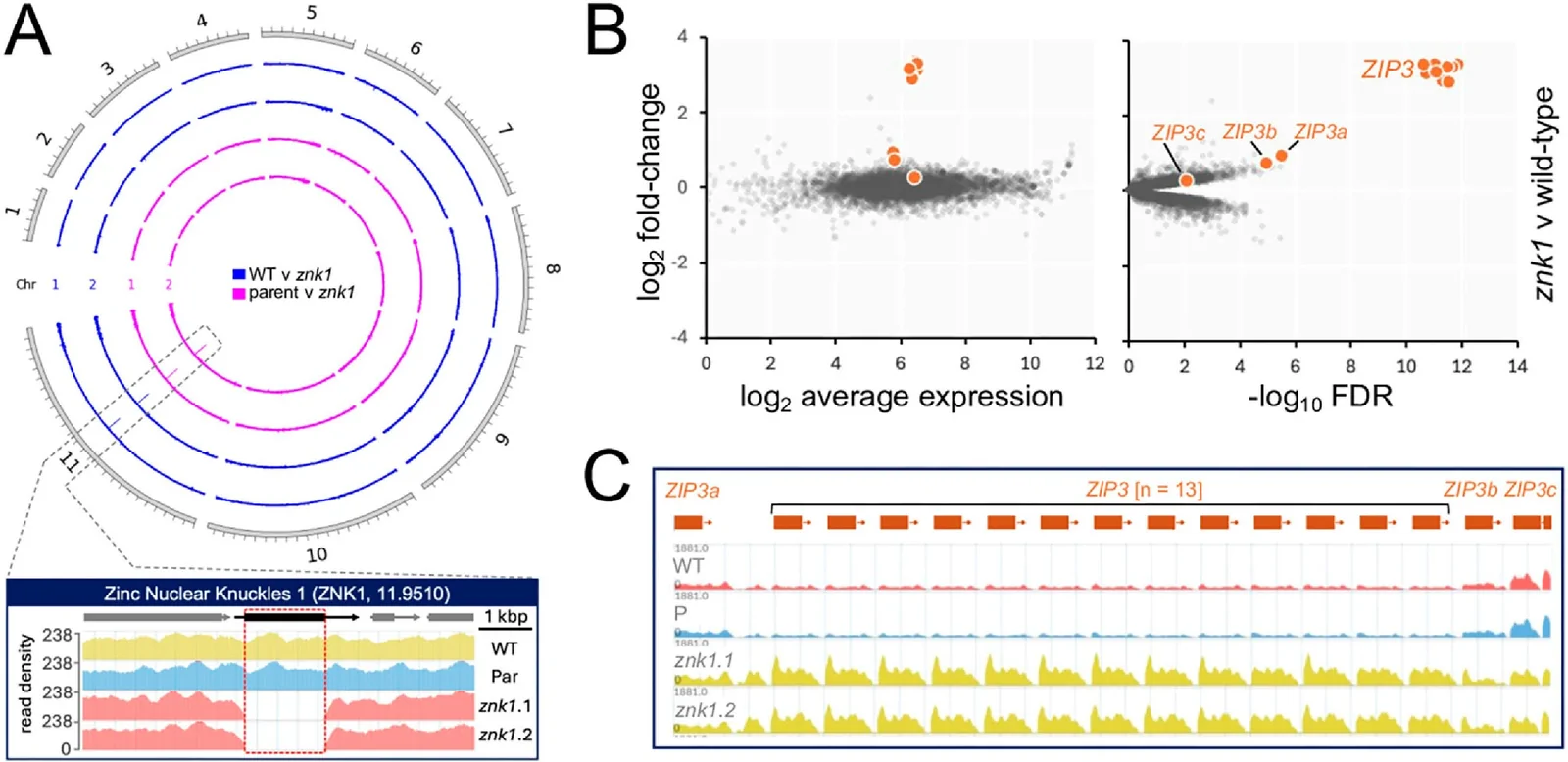
Specific accumulation of *ZIP3* transcripts in *znk1* null cells. (**A**) The circular plot shows whole genome sequencing data for two independent *znk1* null strains compared to WT or parental control strains, revealing specific knockout of the *ZNK1* gene on chromosome 11. The lower, zoomed panel shows precise *ZNK1* deletion. (**B**) RNA-seq analysis comparing *znk1* null and WT cells. *ZIP3* and *ZIP3a-c* transcripts are highlighted. The data represent averages from three independent technical replicates in each case. *n* = 8013, using the 427 experimental strain genome. (**C**) RNA-seq analysis showing the *ZIP3*/*3a-c* locus (37 kbp) in the WT and parental (P) control strains and in the *znk1* null strains.

To determine whether ZNK1 controls the abundance of *ZIP3* and/or other mRNAs, we performed CRISPR–Cas9-mediated knockout of the *ZNK1* gene in *T. brucei* bloodstream-form cells. From two independent transfections, we selected two *znk1* null clones where both *ZNK1* alleles were replaced with a *NEO* cassette, as determined by PCR (Supplementary Fig. S5), and genome sequencing was used to validate specific deletion of the *ZNK1* gene in both strains (Fig. 4A).

To assess the impact of ZNK1 on mRNA abundance, we performed RNA sequencing (RNA-seq) under standard (zinc-sufficient) conditions with parental cells and both *znk1* null strains. RNA-seq reads were mapped to the ‘927’ reference genome initially (Supplementary Data 1, sheets 2–3), revealing excellent correspondence between *znk1* null strains and relatively few transcripts that appeared to be regulated by ZNK1 (Supplementary Fig. S6A). Those transcripts with the most significantly increased abundance in the *znk1* strains were from the *ZIP3* locus (7-fold increase, FDR = 2.8^−10^; Supplementary Fig. S6B); transcripts for the other ZIP, Tb927.5.4140, or for two Golgi-localized putative zinc transporters, Tb927.11.15050 and Tb927.11.1910 [13], failed to register a significant (FDR < 0.001) change in abundance. Gene Ontology profiles for those other transcripts that achieved moderate but significantly (FDR < 0.001) increased expression (n = 167, average 45% increased) yielded terms including ‘nucleoside metabolism’, ‘mRNA binding’, ‘cell cycle’, and ‘transcription’; while ‘metal ion binding’ was associated with those transcripts with significantly (FDR < 0.001) decreased expression (*n* = 126, average 28% decreased; Supplementary Fig. S6C). We suggest that these changes reflect the consequences of increased ZIP3 expression and subsequent increase in intracellular zinc (see below).

RNA-seq reads were then mapped to the experimental ‘427’ strain genome (Supplementary Data 1, sheets 4–5) since tandem gene arrays are generally more reliably assembled in this genome [46]. Indeed, the *ZIP3*-like array comprises five genes in the ‘927’ genome (Tb927.11.8990–Tb927.11.9030) and sixteen genes in the ‘427’ genome (Tb427_110096500–110098000). As above, the analysis confirmed *ZNK1* knockout with 4487 reads mapped to this gene in the parental strain and two reads in the knockout strains. The analysis also again revealed a highly significant increase in *ZIP3* transcript abundance (fold-change = 9.3 ± 0.9, False Discovery Rate [FDR] <1e^−11^) in the *znk1* null strains (Fig. 4B and C); *ZIP3a-c* transcript abundance was also significantly increased, although <2-fold in each case.

### *znk1* null cells accumulate excess zinc and are zinc-sensitive

**Figure 5.**
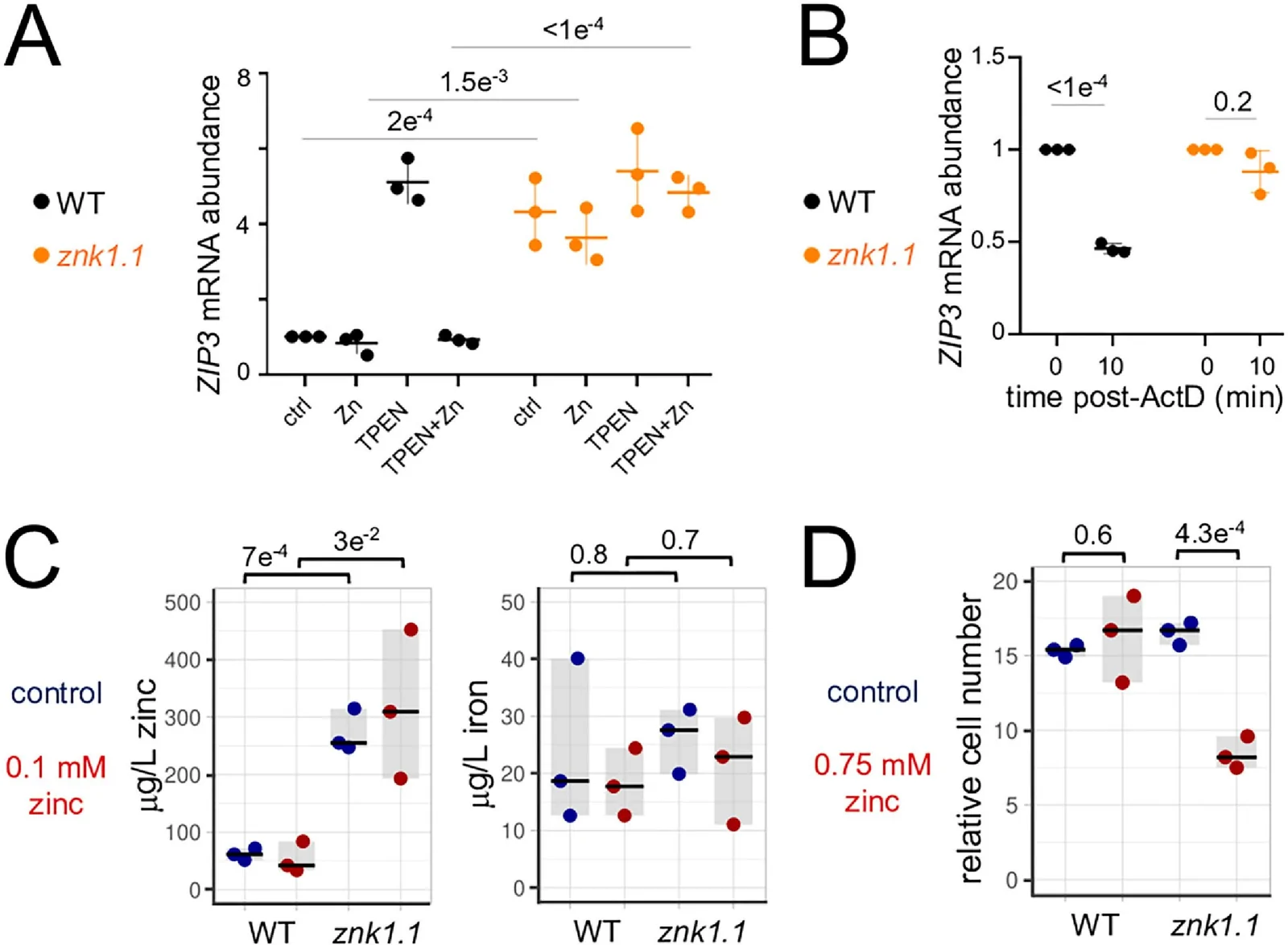
*znk1* null cells accumulate excess zinc and are zinc-sensitive. (**A**) The plot shows RT-qPCR analysis to assess the abundance of *ZIP3* transcripts in WT and *znk1.1* null cells. Other details as in Fig. 1A and B. (**B**) The plot shows RT-qPCR analysis to assess the *ZIP3* transcript after inhibition of transcription with actinomycin D (ActD, 5 µg/ml) in WT and *znk1.1* null cells. Other details as in Fig. 1A and B. (**C**) The plots show total cellular zinc (left-hand plot) or iron (right-hand plot) content in WT and *znk1.1* null cells under standard conditions or following supplementation with 100 µM zinc for 2 h. *n* = 3 technical replicates. (**D**) The plot shows cell density after growth of WT or *znk1.1* null cells with or without excess zinc (0.75 mM) for 24 h. *n* = 3 technical replicates with n > 100 cells in each case. Horizontal lines in panels (C and D) indicate the median and the grey boxes indicate the range of values; *P-*values were calculated using two-sided *t*-tests.

Having established that *ZIP3* transcript negative control was lost in *znk1* null cells, we sought to further characterize the impact of increased *ZIP3* expression in these cells. First, we further validated increased *ZIP3* mRNA abundance in *znk1* null cells using RT-qPCR, showing that *ZIP3* transcripts were significantly elevated in the absence of ZNK1, independent of zinc availability in the culture media (Fig. 5A). Using the same approach, we observed no significant increase in the abundance of *ZIP3a* transcripts (Supplementary Fig. S7A) or transcripts for the other ZIP (Tb927.5.4140) (Supplementary Fig. S7B). As expected, we found *ZIP3* transcripts to be significantly more stable in *znk1* null cells relative to WT controls (Fig. 5B).

Increased ZIP3 expression in *znk1* null cells may lead to an increase in intracellular zinc. This was indeed found to be the case, with intracellular zinc increasing significantly and by >4-fold in *znk1* null cells, regardless of whether the medium was supplemented with 100 µM zinc (Fig. 5C, left-hand panel); while intracellular iron accumulation was notably not significantly different in *znk1* null cells assessed under the same conditions (Fig. 5C, right-hand panel). Consistent with increased accumulation of zinc in *znk1* null cells, the growth of these cells was significantly diminished in 0.75 mM zinc for 24 h, while the growth of WT cells appeared unperturbed under the same conditions (Fig. 5D). The intracellular zinc accumulation phenotype associated with loss of ZNK1-mediated negative control and increased *ZIP3* expression suggested that the ZIP3 transporter does indeed control zinc uptake. The zinc-sensitivity phenotype is likely due to protein mismetallation [2–4] under excess zinc.

### ZNK1 operates via the *ZIP3* 3′-UTR

**Figure 6.**
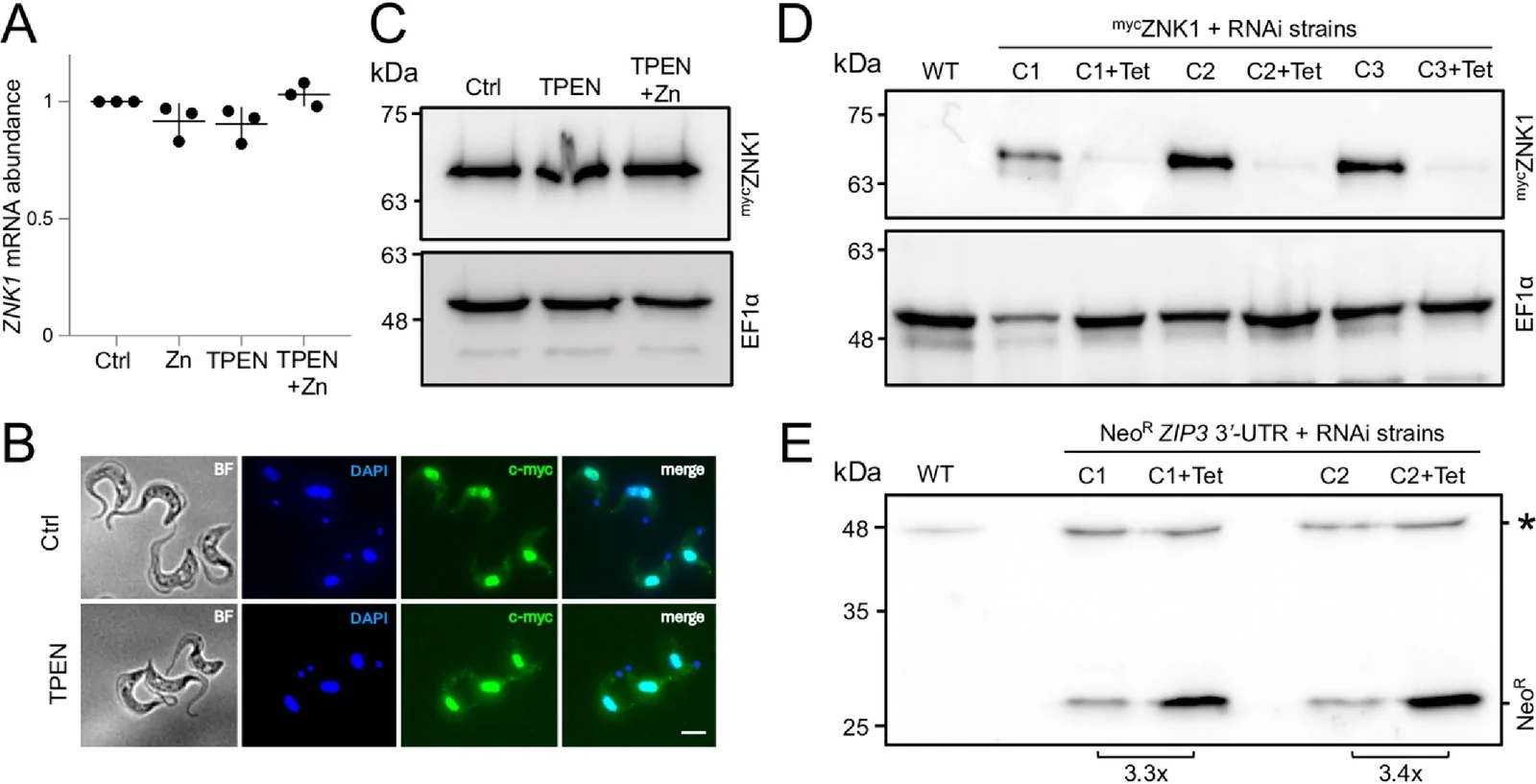
ZNK1 operates via the *ZIP3* 3′-UTR. (**A**) The plot shows RT-qPCR analysis to assess the abundance of *ZNK1* transcripts. Other details as in Fig. 1A and B. (**B**) Immunofluorescence microscopy showing the localization of ^myc^ZNK1 in control (Ctrl) or zinc-depleted (TPEN, 2 h) medium. Scale bar = 5 µm. (**C**) The protein blot shows the expression of ^myc^ZNK1 in control (Ctrl), zinc-depleted (TPEN, 10 µM, 2 h), or zinc-supplemented (TPEN + Zn, 10 µM + 100 µM, 2 h + 1 h) medium. The EF1α panel serves as a loading control. (**D**) The protein blot shows efficient ^myc^ZNK1 knockdown following induction of ZNK1 RNAi using tetracycline (Tet) for 24 h. The EF1α panel serves as a loading control. WT, wild-type, C1–3, clones 1 to 3 represent three biological replicates. (**E**) The protein blot shows expression of the Neo^R^  *ZIP3* 3′-UTR reporter before and after knockdown of ZNK1 for 24 h. A non-specific band (*) serves as a loading control. Relative increase in the Neo^R^ signal following ZNK1 knockdown is indicated below the blot. WT, wild-type, C1–2, clones 1 and 2 represent two biological replicates. *n* = 1 for protein blots in panels (C–E).

To characterize ZNK1 in more detail, we first analysed ZNK1 transcript abundance in various conditions of zinc availability. First, we found that the *ZNK1* transcript is not substantially regulated by zinc concentration in the culture medium (Fig. 6A). We next assembled a bloodstream-form *T. brucei* strain expressing N-terminally c-myc-tagged ZNK1 (^myc^ZNK1) from its native locus. Immunofluorescence microscopy showed that ^myc^ZNK1 was strongly enriched in the bloodstream-form *T. brucei* nucleus (Fig. 6B), and neither ZNK1 subcellular localization (Fig. 6B) nor abundance (Fig. 6C) were substantially changed following zinc chelation with TPEN.

We next used ZNK1 knockdown to determine whether ZNK1 does indeed operate via the *ZIP3* 3′-UTR. A bespoke ZNK1 RNAi construct was used for robust ZNK1 knockdown (Fig. 6D), which led to increased expression of the Neo^R^  *ZIP3* 3′-UTR reporter (Fig. 6E). This result phenocopied zinc limitation and was entirely consistent with identification of ZNK1 in the RIT-seq screen. This also demonstrated that *ZNK1* emerged as a hit in our RNAi screens due to control of the Neo^R^  *ZIP3* 3′-UTR reporter, rather than by increasing G418 resistance by another mechanism, as was thought to be the case for the MFS transporter, for example (see above). Thus, ZNK1 mediates negative regulation via the *ZIP3* mRNA 3′-UTR when zinc availability is sufficient.

### A GU-repeat motif in the *ZIP3* 3′-UTR is implicated in ZNK1-mediated control

**Figure 7.**
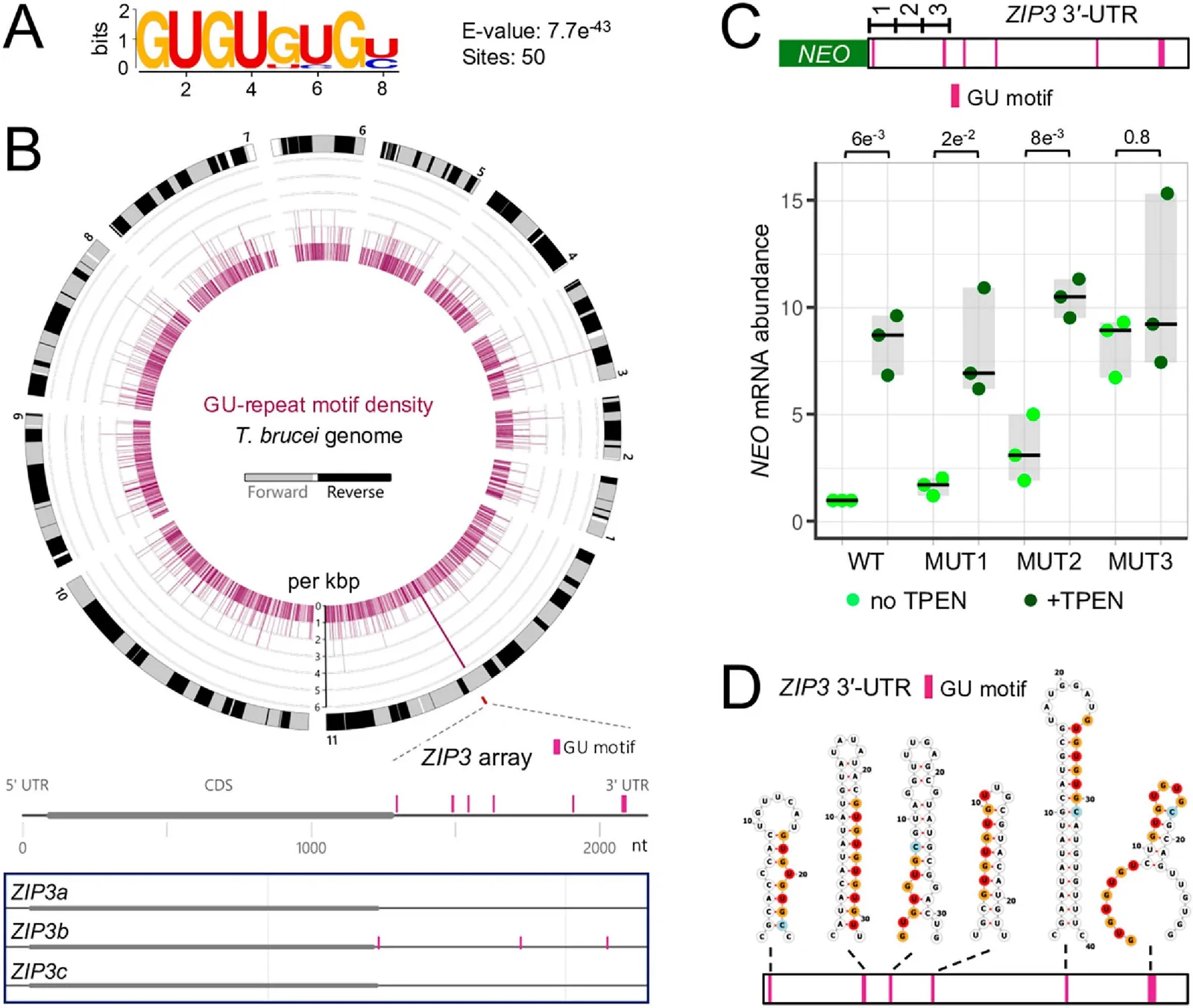
A GU-repeat motif in the *ZIP3* 3′-UTR is implicated in ZNK1-mediated control. (**A**) Motif enrichment analysis revealed a GU-repeat motif in *ZIP3* 3′-UTRs from multiple trypanosomatids. (**B**) Density of the GU-repeat motif across the ‘427’ genome assembly is shown on the circular karyotype plot (inner circle). The outer circle shows chromosomes and polycistronic transcription units. Only the Tb427_020011900 and Tb427_110182200 genes, on chromosomes 2 and 11, register 4 GU-repeat motifs/kbp, while other regions on chromosomes 3, 7, and 8 that register ≥4 GU-repeat motifs/kbp do not appear to encode mRNA. The lower map shows the location of six GU-repeat motifs in the *ZIP3* (Tb427_110096600) transcript, all in the 888-b 3′-UTR. The lower box shows the location of GU-repeat motifs in the *ZIP3a-c* transcripts from genes that flank the *ZIP3* array (Tb427_110096500 and Tb427_110097900–8000). (**C**) The upper map shows the regions replaced with randomized sequence in the *ZIP3* 3′-UTR *NEO* reporter constructs to generate mutants 1–3. The plot shows RT-qPCR analysis to assess the abundance of *NEO* reporter transcripts with wild type (WT) or mutant 3′-UTRs. Reporter strains were grown in zinc-sufficient or zinc-limited medium (10 µM TPEN for 2 h). Data represent fold change in expression relative to the WT control, normalized to β-tubulin transcript abundance. Horizontal lines indicate the median and the grey boxes indicate the range of values. *P*-values were derived using two-way ANOVA tests with Tukey’s multiple comparisons. *n* = 3 technical replicates. (**D**) Each of the GU-repeat motifs in the *ZIP3* 3′-UTR is predicted to be associated with local RNA secondary structure (http://rna.tbi.univie.ac.at/cgi-bin/RNAWebSuite/RNAfold.cgi).

The conserved arrangement of zinc knuckle motifs in ZNK1 suggested a mechanism involving recruitment to *ZIP3* mRNA via binding to a repeated *cis*-acting element. To identify candidate *cis*-acting sequences, we searched for motifs that were over-represented among *ZIP3* ortholog 3′-UTRs from eleven trypanosomatid genomes, including *T. cruzi* and *Leishmania* (Supplementary Data 1, sheet 6). This analysis revealed a GU-repeat motif present in all eleven 3′-UTR sequences (Fig. 7A, E value 7.7^−43^). Although there are many copies of this 8-b GU-repeat motif encoded in the *T. brucei* genome, a genome-wide search revealed the highest density of this motif at the *ZIP3* locus, being present at six locations within each *ZIP3* 3′-UTR (Fig. 7B) and present at three locations within each putative *T. cruzi* and *Leishmania ZIP3* 3′-UTR. Such a repeated *cis*-acting sequence could act synergistically to recruit a multi-zinc knuckle/finger protein such as ZNK1. Notably, the GU-repeat motif is either absent or diminished within *ZIP3a-c* 3′-UTRs (Fig. 7B, lower panel), consistent with <2-fold increased abundance of these transcripts in *znk1* null cells (Fig. 4B and C).

To assess components of the *ZIP3* 3′-UTR required for zinc-dependent regulation, we used the Neo^R^  *ZIP3* 3′-UTR reporter construct described above and derived three mutants (MUT1–3), each with a sequential 75 bp region from the stop-codon proximal end of the 3′-UTR replaced with the same randomized sequence; one GU-repeat motif is replaced in MUT1, no GU-repeat motifs are replaced in MUT2, while a second GU-repeat motif is replaced in MUT3 (Fig. 7C, upper panel). As above, we transformed *T. brucei* cells with the control and mutant reporter constructs and assessed *NEO* reporter mRNA expression by RT-qPCR in the presence or absence of the zinc chelator, TPEN. As expected, the control reporter behaved as described above (Fig. 1C), with *NEO* mRNA expression significantly increased in the presence of TPEN (Fig. 7C). Significantly increased expression in the presence of TPEN was preserved in both mutants 1 and 2, while the response was abrogated in mutant 3 (Fig. 7C). Indeed, *NEO* expression was already elevated in the absence of TPEN in this mutant, indicating loss of 3′-UTR-based negative control. GU-repeat motifs may function by forming mRNA secondary structure, and indeed double-stranded RNA (dsRNA) formation and stem-loop structures are predicted to be associated with each of these motifs in the *ZIP3* 3′-UTR (Fig. 7D). Notably, the GU-repeat replaced in mutant 3 comprises five GU-repeats and is the only motif predicted to be fully incorporated into dsRNA (Fig. 7D). These results implicate GU-repeat motifs in *ZIP3* expression control and are consistent with the view that ZNK1 interacts with these motifs in the *ZIP3* 3′-UTR, potentially impacting ZNK1 recruitment and/or RNase activity.

Together, our results show that *ZIP3* transcripts are relatively stable when zinc supply is limited. In contrast, when zinc supply is sufficient, *ZIP3* transcripts are turned over in a ZNK1-dependent and *ZIP3* 3′-UTR-dependent, likely GU-repeat motif-dependent, manner. Thus, we establish ZNK1 as a zinc-dependent repressor of ZIP3 transporter expression.

## Discussion

Regulation of zinc homeostasis is essential for cellular function and relies on the coordinated activity of zinc transporters and zinc storage [47]. However, the mechanisms by which zinc availability is sensed and translated into changes in transporter expression have remained incompletely characterized in trypanosomatids and in many other organisms. Using 3′-UTR reporter assays, genome-wide RNA interference (RIT-seq) screening [38, 48], and transcriptomics, we identified and characterized *T. brucei* Zinc Nuclear Knuckles 1 (ZNK1), a zinc sensory RNA-binding protein required for zinc-dependent control of the probable *T. brucei* zinc transporter ZIP3. ZNK1 is highly conserved among the trypanosomatids and is therefore likely to be the key post-transcriptional zinc homeostasis regulator in these important parasites.

We first found that *T. brucei ZIP3* mRNA turnover is increased in the presence of zinc, and by a mechanism involving the 3′-UTR. Exploiting this information, we used a drug-resistance marker under the control of the *ZIP3* 3′-UTR as a reporter in a genome-wide RIT-seq screen. The screen identified ZIP transporter knockdowns that reduced zinc availability, leading to activation of the reporter, and also identified the putative regulator, ZNK1. We validated ZNK1 in bespoke RNAi strains and also used CRISPR-Cas9 to knockout *ZNK1*; these cells were defective for zinc-dependent negative control of both *ZIP3* mRNA and the *ZIP3* 3′-UTR reporter.

Trypanosomatid ZNK1 incorporates seven or eight zinc knuckle motifs and one to two CCHC-type zinc finger motifs in the N-terminal region. These tandem zinc-binding motifs are suggestive of a role in zinc sensing [49]. Zinc knuckles are compact Cys/His-rich modules that typically engage single-stranded RNA, often acting as RNA ‘chaperones’ that stabilize transient structures. When present in tandem, they can create an extended, modular binding surface that can recognize both sequence features and local RNA architecture along a 3′-UTR [50]. In trypanosomatid ZNK1, the presence of several knuckle motifs is therefore consistent with high-affinity and selective recognition of *ZIP3* 3′-UTRs. Because coordination of zinc is integral to knuckle folding, changes in metal occupancy are expected to modulate the stability and orientation of the knuckles and, thus, RNA binding.

**Figure 8.**
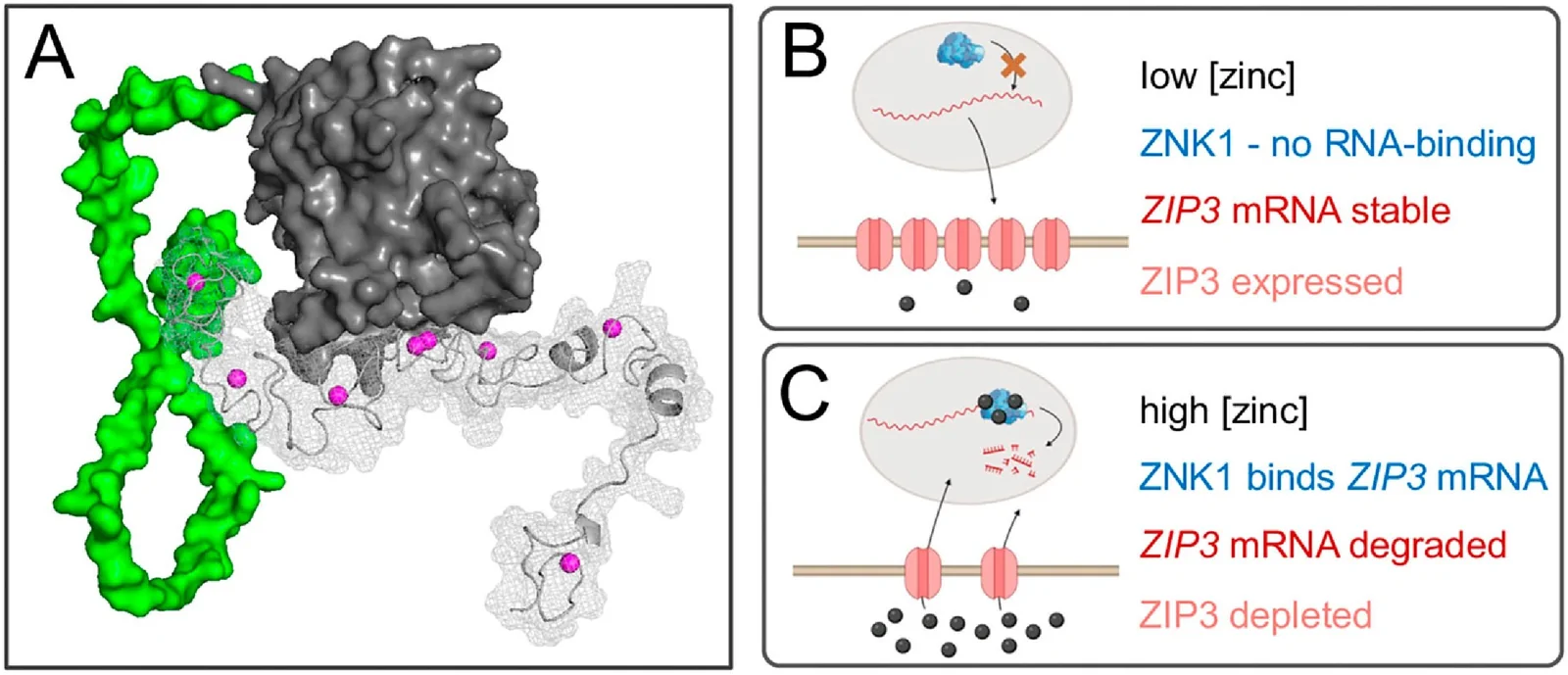
A speculative model for zinc sensing and *ZIP3* mRNA turnover by ZNK1. (**A**) The AlphaFold model (https://alphafoldserver.com/) shows *T. brucei* ZNK1 visualized using PyMOL and showing the zinc knuckle/finger region coordinating eight Zn^2+^ ions (magenta), an unstructured ‘linker’ region (green), and the PIN domain (grey); ipTM = 0.3, pTM = 0.46. We suggest that zinc facilitates binding to *ZIP3* mRNA, thereby recruiting the nuclease domain to its substrate. (**B**) The schematic model indicates ZNK1 remaining inactive in the nucleus in zinc-limiting environments, facilitating plasma membrane-localized ZIP3 expression and zinc uptake. (**C**) The schematic model indicates ZNK1 binding to and degrading *ZIP3* mRNA in zinc-sufficient environments.

The C-terminal portion of ZNK1 encodes an NYN/PIN family, PIN superfamily, putative nuclease domain with conserved acidic residues typical of a metal-dependent catalytic centre [43]. Other PIN-domain proteins in trypanosomatids include RRP44/Dis3 [51, 52], protein*-*only RNase P enzymes [53], and ESB2 [54], which participate in rRNA/tRNA processing or subtelomeric expression-site-associated-gene control, respectively. Another PIN-domain protein, Regnase, negatively controls inflammatory response transcripts in mammals and other metazoa [55]. ZNK1 is a unique member of the NYN/PIN family with an architecture suggestive of endoribonuclease activity coupled to a micronutrient-sensor module. Thus, ZNK1 architecture is entirely consistent with our demonstration of zinc-dependent turnover of *ZIP3* mRNA. An AlphaFold model [56] shows how the putative sensor and RNA nuclease domains in ZNK1 may be connected by a more divergent and unstructured ‘linker’ (Fig. 8A).

*ZIP3* mRNA regulation by ZNK1 is remarkably specific. Nuclear localization of ZNK1 also suggests that *ZIP3* mRNA turnover occurs in the nucleus, possibly immediately following transcription. We suggest a model whereby, under limiting zinc conditions, the zinc-knuckles in ZNK1 fail to fold, and fail to bind *ZIP3* mRNA, which can then be effectively exported from the nucleus and translated, facilitating zinc uptake (Fig. 8B). When ZIP3 transporter activity provides an increasing supply of zinc, the knuckles fold more efficiently such that ZNK1 binds and turns over *ZIP3* mRNA (Fig. 8C). Indeed, we identify and validate a strong candidate GU-repeat motif and *cis*-acting sequence likely required for productive ZNK1-binding. Thus, zinc-dependent changes in the RNA-binding module facilitate highly specific recruitment of this RNase to target substrates. This simple sensing and feedback model likely explains zinc homeostasis control by the conserved ZNK1 RNA-binding protein in the trypanosomatids.

The specificity of ZNK1 for *ZIP3* contrasts with systems in which a single factor coordinates a broad zinc response, such as Zap1 in budding yeast, MTF-1 in mammals, or the uptake regulator Zur in bacteria, which control zinc uptake and storage regulons as well as the expression of additional adaptive proteins at the transcriptional level [7, 57, 58]. In those cases, zinc availability is sensed by DNA-binding transcription factors. Thus, post-transcriptional control in *T. brucei* is distinct and facilitates nutrient-responsive control in the context of widespread polycistronic transcription. Similarly, the *T. brucei* NT8 nucleobase transporter mRNA is repressed by the Purine Response Element Binding Protein (PuREBP)1/2 complex in a 3′-UTR PuRE stem-loop-dependent manner when purines are abundant [59, 60]; although the purine sensing and effector mechanisms are not fully understood. Glucose and amino acid transporters are also subject to 3′-UTR-based post-transcriptional controls in *T. brucei* [61, 62]. ZNK1 therefore extends a paradigm in which trypanosomes implement nutrient sensing through RNA-centric mechanisms, whereby RNA-binding proteins selectively bind regulatory 3′-UTRs, rather than employing transcription factor-based feedback loops. Indeed, the predicted structure of ZNK1 suggests that a single RNA-binding protein can incorporate both sensing and effector functions.

In conclusion, our study reveals a zinc-dependent post-transcriptional mechanism that modulates ZIP transporter expression in *T. brucei*. ZNK1 emerges as a key component of a zinc-responsive circuit linking metal availability to mRNA stability. This work uncovers a new dimension of parasite metal homeostasis and illustrates how eukaryotic cells can repurpose RNA decay machinery to achieve precise adaptation to fluctuating micronutrient availability. ZNK1 conservation suggests that this zinc sensor negatively controls zinc uptake to maintain appropriate zinc supply in the varying host environments encountered by the parasitic trypanosomatids.

## Supplementary Material

gkag877_Supplemental_Files

## Data Availability

The high-throughput sequencing data (RIT-seq, whole genome sequencing, and RNA-seq) have been deposited in the Sequence Read Archive under accession code PRJNA1405336 (https://www.ncbi.nlm.nih.gov/bioproject/PRJNA1405336).
