# Supplementary material for: Zinc-dependent turnover of ZIP3 transporter mRNA by trypanosome ZNK1
Source: Nucleic Acids Res. 2026 Sep 12;54(17):gkag877. doi: 10.1093/nar/gkag877 (PMC13569496; doi:10.1093/nar/gkag877)
Supplement: Supplementary file 1 [file gkag877_supplemental_files.zip › ZNK1 Supplement.pdf]

Zinc-dependent turnover of ZIP3 transporter mRNA by trypanosome ZNK1

Teresa Leão, Anna Trenaman, Michele Tinti, Gustavo Bravo Ruiz, Idálio J. Viegas, Luisa M. Figueiredo, Margarida Duarte, Ana Maria Tomás and David Horn

Supplementary Figures 1-7

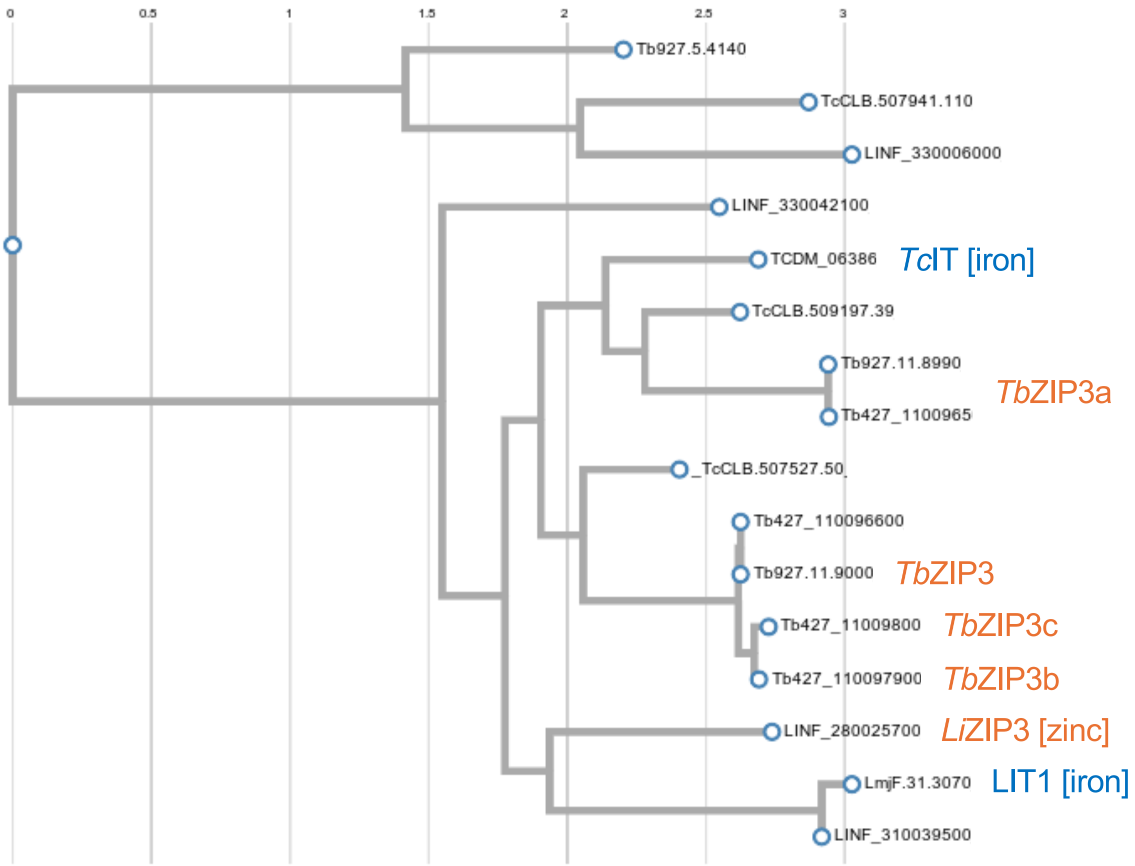

Supplementary Figure 1. Phylogenetic analysis of ZIP proteins in trypanosomatids; *Trypanosoma brucei, T. cruzi, Leishmania infantum* and *L. major*. GeneIDs are indicated and sequences are available from tritrypbd.org. Additional labels refer to genes detailed in the main text, including reported iron transporters. The tree was produced using ETE3.

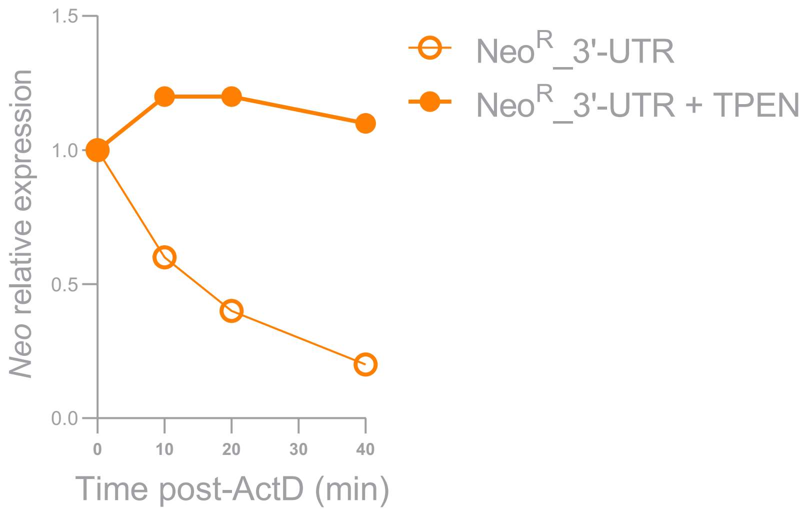

Supplementary Figure 2. RT-qPCR analysis to assess the Neo^R *ZIP3* 3′-UTR reporter transcript after inhibition of transcription with actinomycin D (ActD, 5 μg/mL), in control or TPEN-treated cells. n = 1. Other details as in Fig.1D, except the data were normalised to *VSG* transcript abundance in this case.

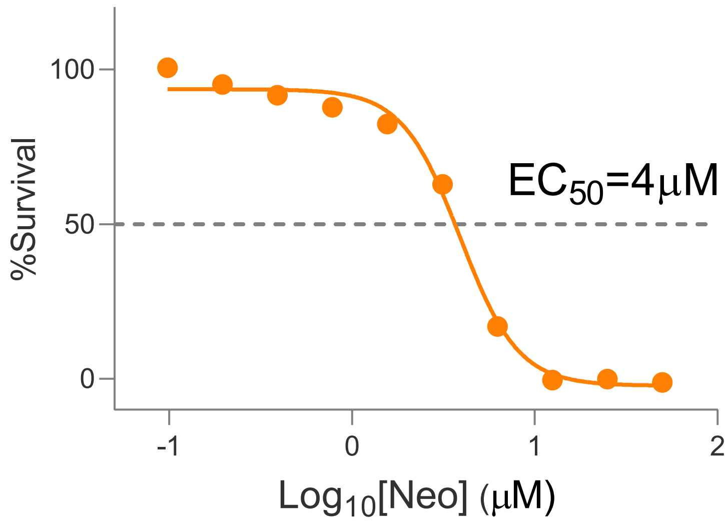

Supplementary Figure 3. Neomycin (G418) dose-response curve for the *T. brucei* Neo[R] *ZIP3* 3′-UTR reporter strain. We used 2 x EC_{50} for RIT-seq library screening to select for loss of *ZIP3* 3′-UTR-based negative control.

A

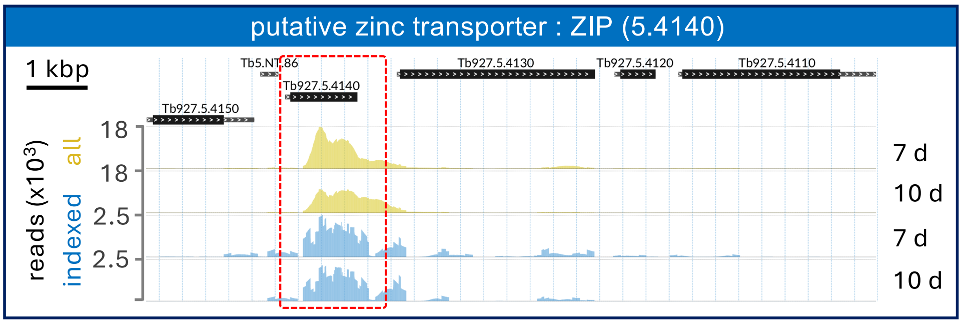

B

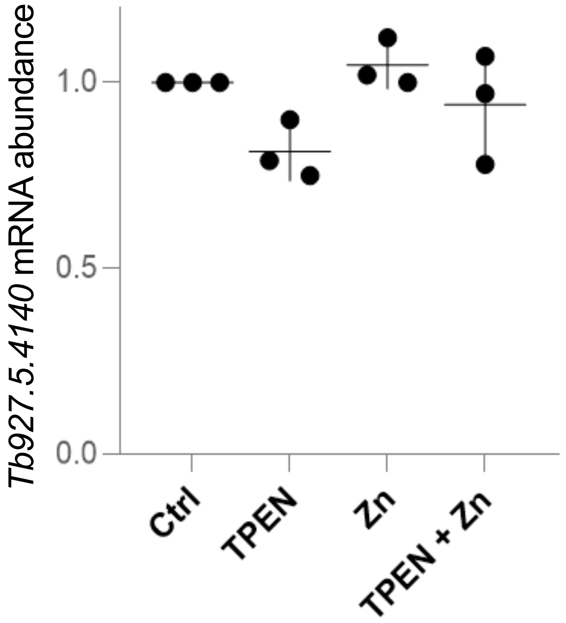

Supplementary Figure 4. RIT-seq identifies another putative *ZIP* (Tb927.5.4140) as a hit. (**A**) The profile shows enrichment of RIT-seq fragments at the 5.4140 locus. Other details as in Fig. 2D. (**B**) RT-qPCR assays were used to assess the abundance of 5.4140 transcripts in *T. brucei* grown in zinc-sufficient or zinc-limiting conditions. Other details as in Fig. 1A.

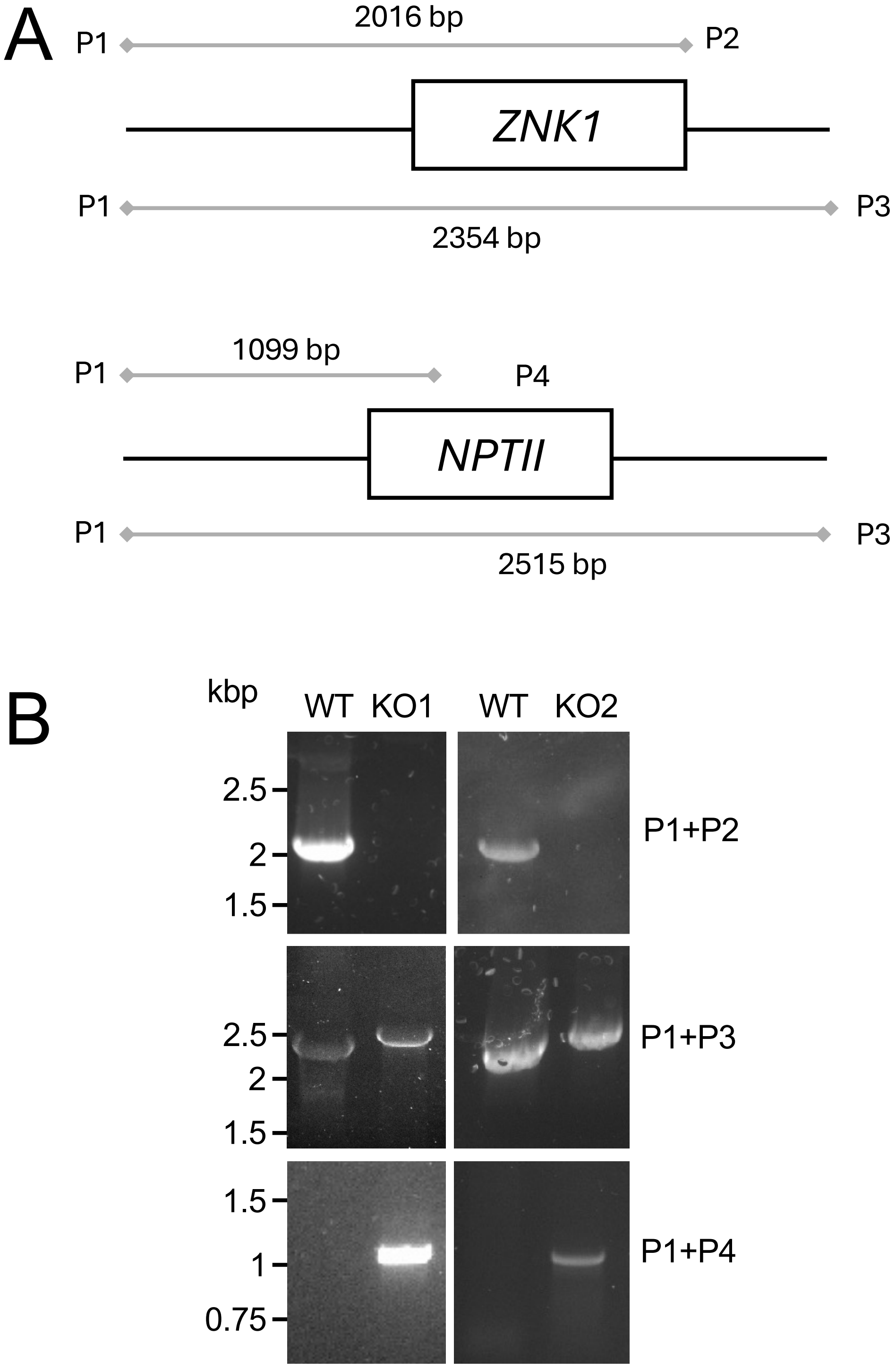

Supplementary Figure 5. Knockout of *T. brucei ZNK1* (**A**) The schematic maps show the *ZNK1* locus before (upper panel) and after (lower panel) CRISPR-Cas9-mediated knockout. Primers used for the PCR assays are shown. (**B**) PCR assays using genomic DNA before and after *ZNK1* knockout; two independent knockout strains were analysed (KO1 and KO2).

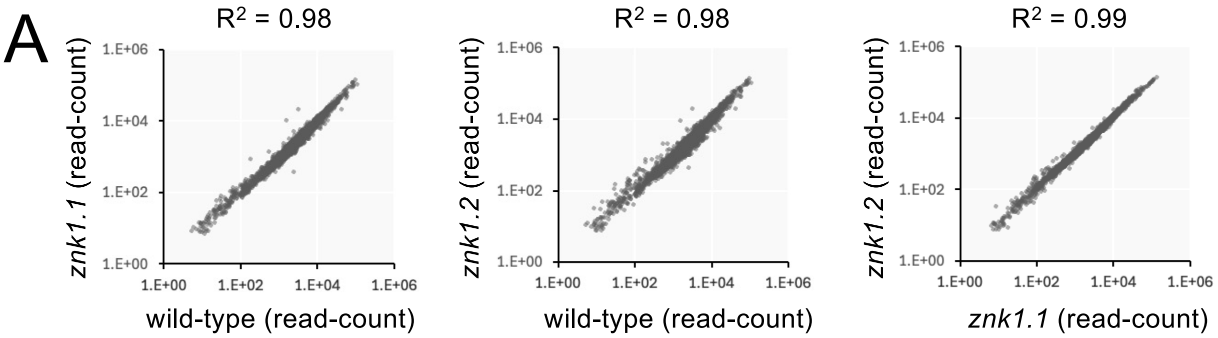

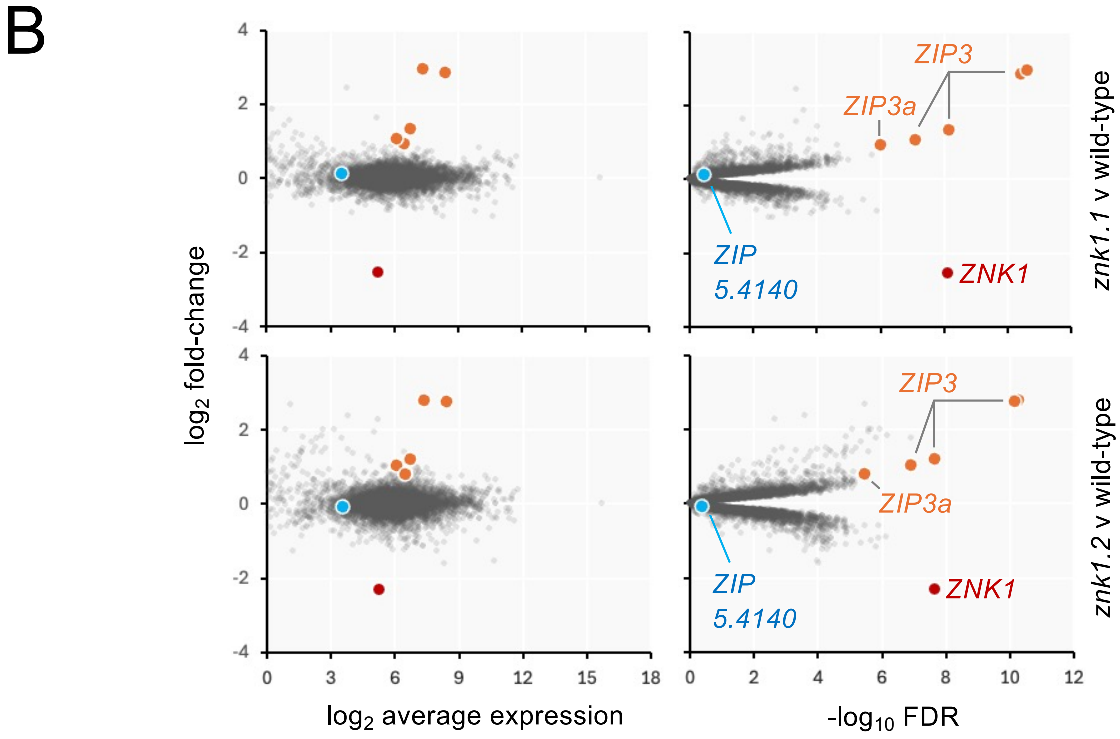

C

Expression significantly increased (FDR > 3) in *znk1* cells (n = 167)						
GO ID	GO Term	Genes in the bkgd with this term	result with this term	Percent of bkgd genes in your result	Fold enrichment	P-value
GO:0009116	nucleoside metabolic process	33	7	21.2	12.04	1.33E-06
GO:1901657	glycosyl compound metabolic process	36	7	19.4	11.04	2.49E-06
GO:0003729	mRNA binding	249	14	5.6	3.19	1.21E-04
GO:0010468	regulation of gene expression	600	23	3.8	2.18	3.15E-04
GO:0000278	mitotic cell cycle	151	10	6.6	3.76	3.21E-04
GO:0019222	regulation of metabolic process	687	25	3.6	2.07	3.73E-04
GO:0019752	carboxylic acid metabolic process	188	11	5.9	3.32	4.70E-04
GO:0006357	regulation of transcription by RNA polymerase II	39	5	12.8	7.28	5.64E-04
GO:0020015	glycosome	185	10	5.4	3.07	1.56E-03
GO:0016570	Histone modification	51	5	9.8	5.57	1.94E-03
GO:0140098	catalytic activity, acting on RNA	231	10	4.3	2.46	7.58E-03
GO:0120119	flagellum attachment zone	102	6	5.9	3.34	9.11E-03

Expression significantly decreased (FDR >3) in *znk1* cells (n = 126)						
GO ID	GO Term	Genes in the bkgd with this term	result with this term	Percent of bkgd genes in your result	Fold enrichment	P-value
GO:0046872	metal ion binding	620	17	2.7	2.15	2.13E-03

Supplementary Figure 6. RNA-seq analysis using the '927' reference genome and comparing *znk1* null and wild-type cells; see Supplementary Data 1 (sheets 2-3). (**A**) The plots show pairwise comparisons and reveal excellent correspondence between znk1 null strains. The data represent averages from three independent technical replicates in each case. n = 8186 (8065 from the '927' reference strain and Variant Surface Glycoprotein [VSG] expression sites from the experimental 427 strain; 101 Expression Site-Associated Genes and 20 VSGs). (**B**) The plots show fold-change and statistical analyses for both the *znk1.1* and *znk1.2* null and wild-type cells. *ZNK1, ZIP3, ZIP3a* and control *ZIP* (*5.4140*) transcripts are highlighted. (**C**) Gene Ontology analysis highlighting terms enriched for transcripts that displayed increased or decreased expression in both *znk1.1* and *znk1.2* strains.

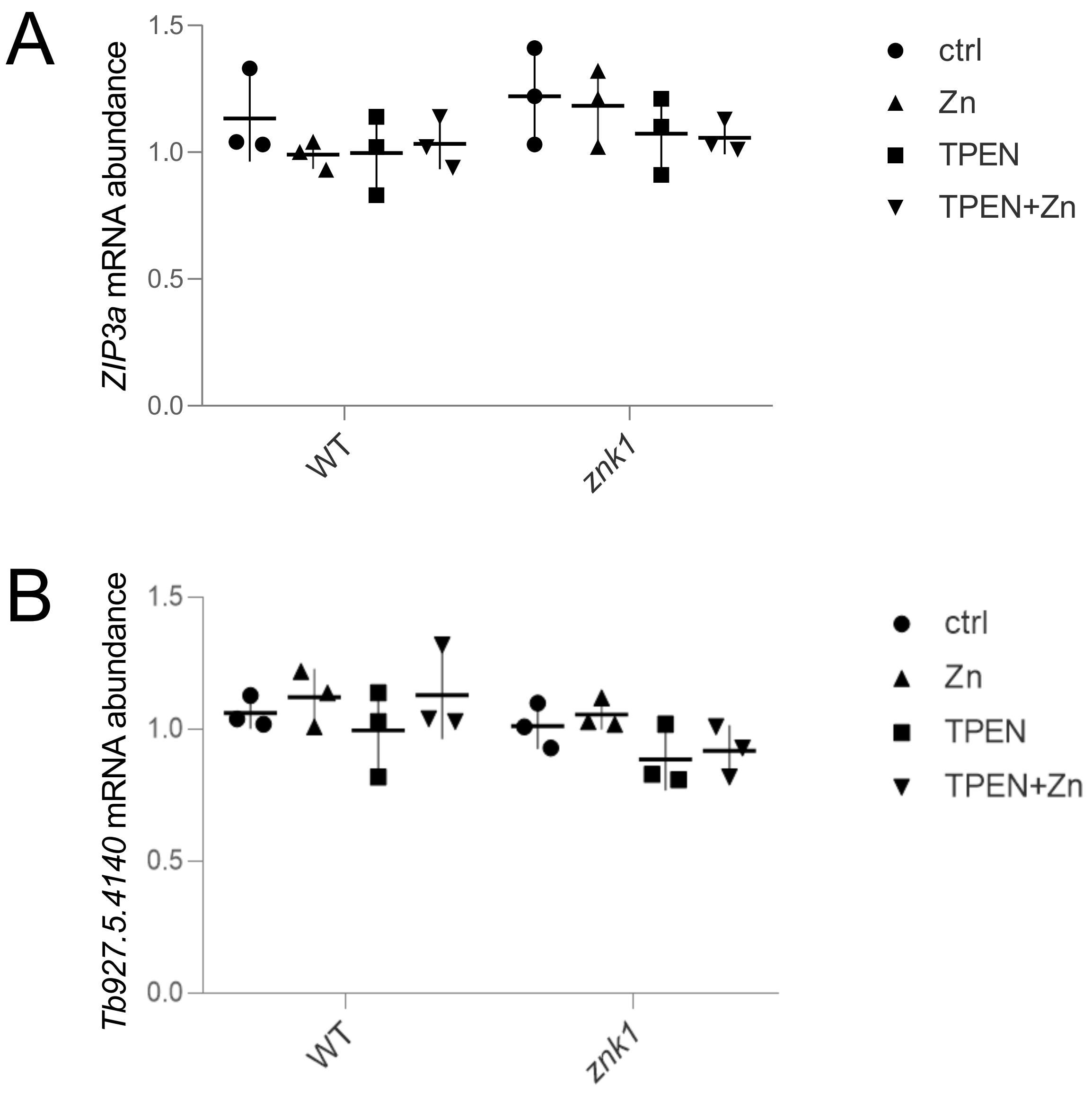

Supplementary Figure 7. RT-qPCR assays were used to assess the abundance of putative ZIP transcripts; *ZIP3'* (**A**) or 5.4140 (**B**) in wild-type or *znk1*-null *T. brucei* grown in zinc-sufficient or zinc-limiting conditions. All *p*-values were >0.05. Other details as in Fig. 1A.
